# A thioether additive as an interfacial regulator for ultra-stable lithium-metal batteries

**DOI:** 10.1093/nsr/nwaf259

**Published:** 2025-06-30

**Authors:** Xiaosong Xiong, Shuanglong Xu, Wenjie Zhang, Qiao Qiao, Yuan Ma, Yiren Zhong, Xin-Bing Cheng, Jiarui He, Zhi Zhu, Faxing Wang, Tao Wang, Yuping Wu

**Affiliations:** Confucius Energy Storage Lab, School of Energy and Environment & Z Energy Storage Center, Southeast University, Nanjing 211189, China; Confucius Energy Storage Lab, School of Energy and Environment & Z Energy Storage Center, Southeast University, Nanjing 211189, China; Confucius Energy Storage Lab, School of Energy and Environment & Z Energy Storage Center, Southeast University, Nanjing 211189, China; School of Chemistry and Molecular Engineering, Nanjing Tech University, Nanjing 211816, China; Confucius Energy Storage Lab, School of Energy and Environment & Z Energy Storage Center, Southeast University, Nanjing 211189, China; Confucius Energy Storage Lab, School of Energy and Environment & Z Energy Storage Center, Southeast University, Nanjing 211189, China; Confucius Energy Storage Lab, School of Energy and Environment & Z Energy Storage Center, Southeast University, Nanjing 211189, China; Confucius Energy Storage Lab, School of Energy and Environment & Z Energy Storage Center, Southeast University, Nanjing 211189, China; Confucius Energy Storage Lab, School of Energy and Environment & Z Energy Storage Center, Southeast University, Nanjing 211189, China; Confucius Energy Storage Lab, School of Energy and Environment & Z Energy Storage Center, Southeast University, Nanjing 211189, China; Confucius Energy Storage Lab, School of Energy and Environment & Z Energy Storage Center, Southeast University, Nanjing 211189, China; Confucius Energy Storage Lab, School of Energy and Environment & Z Energy Storage Center, Southeast University, Nanjing 211189, China

**Keywords:** lithium-metal batteries, electrolyte additive, carbonate electrolyte, thioether, 1,3-dithiane

## Abstract

Rapid inactive lithium accumulation and severe lithium dendrite growth critically limit the cycle life of metallic lithium anodes. Herein, cyclic thioether 1,3-dithiane is reported as a novel electrolyte additive for fabricating ultra-stable lithium-metal batteries. Through the preferential decomposition of 1,3-dithiane additive and PF_6_^−^ anion ions, robust inorganic-rich electrode interphases could be generated at both the anode and the cathode, which is conducive to enhanced kinetics and structural stability of the electrode interface, endowing alleviated active lithium loss and dendrite-free lithium deposition. Moreover, 1,3-dithiane would react with lithium alkylide to reduce the organic component in solid–electrolyte interphases and improve the ability of solvents to resist nucleophilic attack. Consequently, the assembled Li//LiFePO_4_ full cells with 2.0 wt% of the 1,3-dithiane-containing electrolyte exhibit a significantly improved capacity retention of 83.6% after 3300 cycles at a current rate of 1.0 C, which highlights that the 1,3-dithiane additive could induce a long-lifespan lithium-metal battery.

## Teaser

Unlock Li-metal battery longevity! A novel cyclic dithiane additive fortifies solvents shield and forges inorganic-rich electrode interphase for unprecedented Li-metal battery stability.

## INTRODUCTION

Characterized by a high specific capacity (3860 mAh g^−1^) and the lowest electrochemical potential (−3.04 V vs. standard hydrogen electrode), metallic lithium has been regarded as one of the most attractive anode materials for constructing high-energy lithium-metal batteries (LMBs) by replacing the conventional commercial graphite (372 mAh g^−1^) [1]. However, the practical deployment of LMBs faces formidable challenges due to the limited cycling lifespan arising from the rapid depletion of the active lithium and electrolytes. Typically, metallic lithium with high reactivity would react with the electrolyte to form solid–electrolyte interphases (SEIs) containing inorganic components of Li_2_CO_3_, Li_2_O, and organic components of lithium alkyl carbonates, lithium alkoxides, which play an important role in passivating the interface and regulating the interfacial electrochemical behavior [2]. Conventional carbonate-based electrolytes, despite their high oxidation stability, are incompatible with metallic lithium and fail to establish a stable SEI due to the inefficiency of the outer porous structure of the alkyl lithium in separating the electrolyte from the lithium metal, leading to irreversible active lithium loss during the continuous fracture and reformation of the as-formed SEI [3]. Furthermore, aggravated by the infinite volumetric change upon the lithium-deposition/stripping process, the irreversible consumption of active lithium ions and dendritic lithium deposition induced by the poor SEI mean that the assembled LMBs have unsatisfactory coulombic efficiency (CE), rapid battery failure and associated potential safety issues [4,5].

Accordingly, designing a stable SEI is crucially important to ensure highly reversible metallic lithium for long-life LMBs. As the liquid electrolyte plays a pivotal role in determining the intrinsic properties and evolution of the SEI, electrolyte formulation optimization, such as high-concentration electrolytes [6,7], multi-solvent compounding [8,9], dual-salt and multi-salt electrolytes [10,11], new electrolyte component designing [12,13] and functional additives [14,15], has been widely explored to fine-tune the SEI chemistry in LMBs. Among these, the application of electrolyte additives is the most economical and practical strategy. A generally accepted point is that an inorganic-rich interface may boost interface mechanical stability and facilitate Li transport kinetics [16]. As a classic and excellent SEI component, LiF species is expected to alleviate the side reactions by passivating the anode and inducing a dendrite-free Li deposition morphology ascribed to its high interface energy for lithium. Notably, the LiF-abundant layer needs to be finely controlled to prevent degradation due to the relatively low lithium-diffusion kinetics of LiF [17]. Similarly, Li_2_S species-rich interfaces are also demonstrated to derive a stable, protective and high Li^+^ conductive interface, protecting the fragile electrode interface from continuous side reactions [18]. The carbonyl carbon atoms in carbonate solvents are particularly susceptible to decomposition due to ongoing nucleophilic attacks [19]. This leads to the accumulation of a significant number of solvent decomposition products on the electrode surface, which obstructs lithium-ion conduction, increases battery polarization and exacerbates the growth of lithium dendrites. Therefore, interrupting the side reactions of the solvents on the surface of the lithium-metal anode and modifying the resistance of the solvents to nucleophilic attacks may be an effective strategy to eliminate these continuous nucleophilic reactions, ultimately preventing the ongoing solvent decomposition product accumulation of the electrode interface. The development of a multifunctional additive that can simultaneously facilitate the formation of an inorganic-rich SEI, promote the formation of anion-derived films and enhance resistance to solvent decomposition is of great significance for further stabilizing the electrode interface.

**Figure 1.**
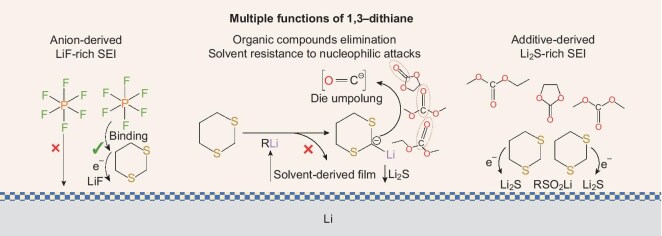
Schematic illustration of the mechanism of 1,3-dithiane additive in electrolyte structure and deposition effect in LMBs.

Herein, a high-sulfur-content electrolyte additive, 1,3-dithiane, is reported for highly-stable LMBs (Fig. 1). Ascribed to a lower lowest unoccupied molecular orbital (LUMO) and a higher highest occupied molecular orbital (HOMO) energy level of the additive compared with the electrolyte solvent, adding 2.0 wt% of 1,3-dithiane into a carbonate electrolyte was proven to promote the preferential formation of an S-rich electrode interface on the electrodes simultaneously (Li_2_SO_3_, C–S species for cathode, Li_2_S for anode). Moreover, the strong interaction between the 1,3-dithiane adsorbed on the lithium-metal anode and the PF_6_⁻ facilitates the decomposition of the PF_6_⁻ anions at the interface, resulting in the formation of a LiF-rich SEI. The inorganic-rich SEI would enhance the kinetics of the lithium-cation transfer and improve the stability of the electrode interface during prolonged cycling. Additionally, the hydrogen atom on the 2-methylene group of the 1,3-dithiane exhibits high acidity, enabling the formation of 2-lithium-1,3-dithiane when it reacts with strong bases such as alkyl lithium, which serves as an intermediate in the synthesis of lithium alkoxy. The reaction effectively inhibits the alkyl lithium from further forming organic components in the SEI. Simultaneously, 2-lithium-1,3-dithiane decomposes on the surface of the lithium metal, resulting in the formation of a sulfur-rich SEI, thereby transforming undesirable organic components of the SEI into S-containing species. Additionally, acting as a polarity-reversal component, 2-lithium-1,3-dithiane enhances the resistance of carbonate solvents to nucleophilic attack, reduces solvent decomposition at the interface and minimizes the formation of organic components within the SEI. The intrinsic high sulfur content (53.5 wt%) of the additive further contributes to its long-term effectiveness. As a result, uniform and dendritic-free lithium deposition is achieved and Li//LiFePO_4_ (LFP) full cells demonstrate remarkable performance, retaining 83.6% of the initial capacity over 3300 cycles at 1.0 C with the 1,3-dithiane-modified electrolyte. Notably, promising performance was further demonstrated by using an Ah-level pouch cell. Compared with the cell with the conventional carbonate electrolyte, the practical Li//LFP pouch cell with the modified electrolyte achieves a 10-times increase in the cycling lifespan with a capacity-retention rate of 93.1% after 150 cycles. This work not only presents a novel cyclothioether electrolyte additive for tailoring the SEI of lithium-metal anodes (LMAs), but also offers a new approach for modifying SEI components.

## RESULTS AND DISCUSSION

### Properties of the 1,3-dithiane additive

**Figure 2.**
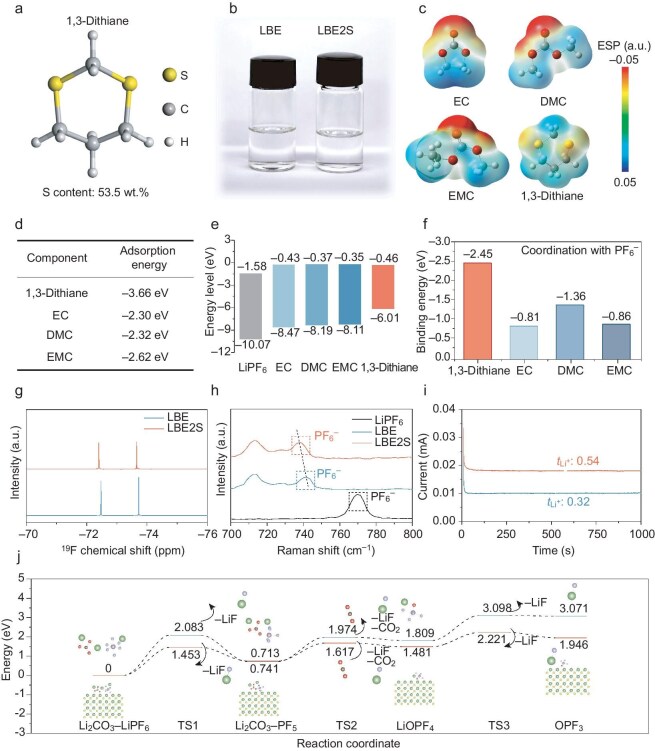
Physicochemical properties of 1,3-dithiane and the corresponding electrolyte. (a) Structural formula of 1,3-dithiane. (b) Photographs of LBE and LBE2S. (c) ESP mapping of EC, DMC, EMC and 1,3-dithiane. (d) Adsorption energies of representative solvents and anions on lithium-metal anode. (e) Electrolyte solvents and solutes LUMO–HOMO energy level. (f) Binding energies of representative solvents and 1,3-dithiane with PF_6_^−^. (g) ^19^F NMR spectra of the LBE and LBE2S. (h) Raman spectra of P–F bond in LBE and LBE2S. (i) Chronoamperometry profiles for the lithium symmetrical cells with LBE and LBE2S. (j) Energy diagrams for the reaction of LiPF_6_ and Li_2_CO_3_ with and without 1,3-dithiane-derived SEI.

The baseline carbonate electrolyte is composed of 1.0 mol L^−1^ of lithium hexafluorophosphate (LiPF_6_) in ethylene carbonate (EC):dimethyl carbonate (DMC):ethyl methyl carbonate (EMC) (1:1:1 by weight ratio) and labeled as LBE. The 1,3-dithiane additive (Fig. 2a and Fig. S1) manifests as a white powder with an extremely high sulfur content of 53.5 wt%, which is nearly double those of conventional sulfur-containing additives (Table S1). A modified carbonate electrolyte was prepared by adding 1,3-dithiane into the LBE. The optimal ratio of the additive addition was determined as 2.0 wt% corresponding to the maximum Li plating/stripping CE and was denoted as LBE2S (Fig. S2). The LBE2S exhibits a clear and transparent appearance, which displays no obvious color difference (Fig. 2b). The wettability of the electrolyte was evaluated by comparing the contact angle on the polypropylene (PP) separator (Fig. S3). The similar contact angles of the LBE2S and LBE electrolytes indicate that the modified electrolyte retains the advantage of the carbonate electrolyte for membrane infiltration, which is conducive to maintaining the rapid ion transport of the electrolyte. The ionic conductivity of the electrolyte at room temperature (∼28°C) following the introduction of the additive is ∼15.3 mS/cm (Fig. S4). Obviously, the introduction of additives did not significantly diminish the high-ionic-conductivity characteristics of the carbonate electrolyte. Furthermore, regarding the long-term storage stability of the electrolyte, we observed that, after 30 days of storage, the appearance of the electrolyte showed no noticeable changes, remaining clear and transparent (Fig. S5a). Raman spectroscopy characterization also revealed that the characteristic peaks of the electrolyte components did not change significantly (Fig. S5b), confirming the high stability of the electrolyte.

The electrostatic potential (ESP) in Fig. 2c shows that the negative charge of the EC, DMC and EMC solvents is predominantly concentrated on the oxygen atoms involved in double bonds, indicating a preferential bonding between lithium cations and O atoms, whereas 1,3-dithiane shows much fewer negative charges near its sulfur atoms, implying its weaker coordination with lithium cations [20].

The preferential adsorption behavior of the 1,3-dithiane additive on the lithium metal was evaluated by using adsorption energy calculation (Fig. 2d and Fig. S6). The lowest adsorption energy of the 1,3-dithiane additive (−3.66 eV) on the LMA facilitates its attachment to the anode, ensuring the effective occurrence of subsequent redox reactions [21]. The components of the electrode interfacial layer highly depend on the LUMO and HOMO energy levels of the electrolyte components. Specifically, 1,3-dithiane possesses the lowest LUMO energy level (−0.46 eV) compared with the EC, DMC and EMC solvents (−0.43, −0.37 and −0.35 eV, respectively) in the LBE electrolyte, indicating its preferential reduction at the SEI (Fig 2e and Fig. S7) [22]. Additionally, the higher HOMO energy level of 1,3-dithiane (−6.01 eV) implies its comparatively inferior resistance to oxidation, which can be supported by the slightly lower decomposition voltage (4.52 V) observed in the linear sweep voltammetry curve (Fig. S8). These findings indicate that 1,3-dithiane was preferentially oxidized and reduced on the cathodes and anodes, respectively, and participated in constructing a robust inorganic-rich electrode interface. The reactivity of the S atom in 1,3-dithiane was evaluated by using the Fukui function (Fig. S9 and Table S2). Both the electrophilicity and the nucleophilicity of the S2 atom exhibit the highest values, indicating that it has the strongest activity within the 1,3-dithiane structure and its preferential involvement in the formation of sulfur-rich species in the cathode–electrolyte interphase (CEI) and SEI [23].

Furthermore, the binding energies of PF_6_^−^ with various electrolyte components were examined (Fig. 2f). The binding energy of PF_6_^−^ with 1,3-dithiane is significantly higher than those of PF₆⁻ with the solvents, which can be attributed to the stronger interactions between the hydrogen and fluorine atoms. Nuclear magnetic resonance (NMR) tests were conducted to demonstrate the strong binding of 1,3-dithian to PF_6_^−^ (Fig. 2g). In the ^19^F spectra, a low field chemical shift of 0.109 and 0.161 ppm occurred in LBE2S, indicating that the coordination of PF_6_^−^ and 1,3-dithiane leads to the weakening of the coupling of Li^+^ and PF_6_^−^. Furthermore, Raman spectra reveal that the band of 740 cm^−1^, which corresponds to the symmetrical stretching vibration mode of PF_6_^−^, exhibits a red shift upon the introduction of 1,3-dithiane (Fig. 2h). This indicates an increased distance between PF_6_^−^ and Li^+^. The phenomenon is attributed to the weakening of the coulombic interaction between PF_6_^−^ and Li^+^ with additives. These results clearly demonstrate the strong interaction between the anions and the additive. In addition, the additive also exhibits a non-negligible effect on the ion-transport behavior of the electrolyte. After the introduction of the 1,3-dithiane additive, owing to the strong interaction between the additive and the anion (Fig. 2i) and the weak interaction with the lithium ion, the migration number of the Li^+^ increases from 0.32 to 0.54, which will be conducive to a decrease in polarization and further enhance the rate performance of LMBs [24]. Besides, anions were attracted to the anode interface through the strong interaction between the anions and the additive, promoting the formation of anion-derived films within the SEI. Figure 2j depicts the reaction pathway and associated energy barriers involved in the interaction between LiPF_6_ and Li_2_CO_3_ to form LiF, analysed in both the presence and the absence of a Li_2_S-rich SEI [25]. The presence of Li_2_S significantly reduces the maximum energy barriers associated with the release of LiF during the LiPF_6_–Li_2_CO_3_ reaction. This indicates that the decomposition of PF_6_^−^ to generate LiF is facilitated by the SEI derived from 1,3-dithiane. The inorganic-rich SEI, comprising Li_2_S and LiF, provides improved chemical and mechanical stability, which is essential for inhibiting ongoing parasitic side reactions at the interface between the electrolyte and the lithium anode.

Additionally, the hydrogen atom on the 2-methylene group of 1,3-dithiane exhibits high acidity, facilitating the formation of 2-lithium-1,3-dithiane when it reacts with strong bases, such as alkyl lithium, which serves as an intermediate in the formation of lithium alkoxy [26]. The reaction effectively prevents alkyl lithium from further generating organic components in the SEI (Fig. S10). Additionally, the low LUMO of 2-lithium-1,3-dithiane allows its preferential decomposition to form an S-rich SEI (Fig. S11), thereby realizing the transformation of undesirable organic components into S-containing species. Additionally, the strong Lewis acidic lithium center in 2-lithium-1,3-dithiane and the Lewis basic carbonyl oxygen in solvent molecules exhibit complementary electronic characteristics, driving their coordination through acid–base interactions. This complexation facilitates pseudo-conjugative interactions between the sulfur atoms of the dithiane ring and the adjacent carbonyl carbon in the solvent molecules. Such electron delocalization induces a polarity inversion (umpolung) at the solvent's carbonyl carbon, transforming its electronic nature from electrophilic to nucleophilic [27]. Consequently, 2-lithium-1,3-dithiane could enhance the resistance of carbonate solvents to nucleophilic attack, reduce solvent decomposition at the interface and minimize the formation of organic components within the SEI.

Charge analysis revealed that the carbonyl carbon atoms in the carbonate solvent molecules exhibit a reduced electron density (from 0.722 to 0.259 for EC, 0.726 to 0.255 for DMC and 0.717 to 0.260 for EMC) in the presence of 2-lithium-1,3-dithiane (Table S3). This charge redistribution indicates a ‘polarity-reversal’ effect, in which the electrophilicity of the carbonyl carbon is suppressed, rendering it less susceptible to nucleophilic attack by alkyl lithium species.

Besides, the LUMO energy levels of EC, DMC and EMC were calculated to have increased significantly (from −0.43 to −0.26 eV for EC, from −0.37 to −0.27 eV for DMC and from −0.35 to −0.26 eV for EMC) when interacting with 2-lithium-1,3-dithiane (Fig. S12). Elevated LUMO levels imply reduced reducibility of the solvents, further corroborating their enhanced resistance to nucleophilic degradation.

### Superior cycling reversibility

**Figure 3.**
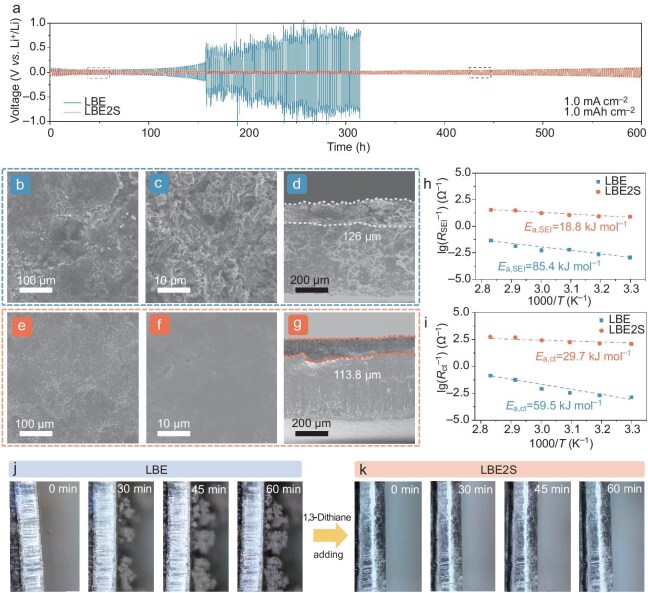
Effect of 1,3-dithiane on performance of lithium anode. (a) Cycling performance of symmetrical cells at 1.0 mA cm^−2^ and 1.0 mAh cm^−2^. Lithium-deposition morphology of lithium symmetrical cells cycled in (b–d) LBE and (e–g) LBE2S. Lithium-cation charge transfer (h) and lithium-cation transportation through (i) SEI. *In situ* optical microscopy visualization of lithium deposition in (j) LBE and (k) LBE2S at 1.0 mA cm^−2^ for 1 h.

The effect of 1,3-dithiane electrolyte additive on enhancing the cycling reversibility of the LMA was assessed by measuring the CE of lithium deposition/stripping in Li//Cu half cells by using a modification of Aurbach's method [28]. The half-cell containing the LBE2S electrolyte demonstrated a higher CE of 97.70% compared with the cell with LBE (95.72%) (Fig. S2), indicating improved stability of the LMA facilitated by the 1,3-dithiane additive. The performance of 1,3-dithiane in repeated lithium deposition/stripping was further evaluated by using lithium symmetrical cells. Rate measurement was first applied to investigate the stability of the SEI under high current densities. Conducted with 0.1, 0.2, 0.3, 0.5, 1.0, 2.0 and 5.0 mA cm^−2^ and then back to 3.0, 2.0 and 1.0 mA cm^−2^ (Fig. S13), the symmetrical cell fabricated with the LBE2S electrolyte reveals superior rate capability with lower overpotential values and a stable voltage plateau at 5.0 mA cm^−2^. In strong contrast, the cell with the LBE electrolyte suffers from voltage fluctuation at only 3.0 mA cm^−2^. The remarkable cycling stability further verifies that 1,3-dithiane effectively stabilizes the electrode interface and inhibits lithium dendrite formation even during high-rate lithium-deposition/stripping processes, owing to the superior SEI properties and enhanced lithium-cation transfer kinetics. Furthermore, at 1.0 mA cm^−2^ and 1.0 mAh cm^−2^ (Fig. 3a), the cell with LBE2S provides stable cycling for >600 h with a low overpotential of ∼46 mV (Fig. S14). In contrast, the cell with the LBE electrolyte failed after 158 h, as indicated by a sudden increase in the fluctuating voltage hysteresis, which can be mainly attributed to the massive consumption of the electrolyte and the accumulation of dead lithium due to the continuous damage and reestablishment of the electrode interface in the carbonate electrolyte [29,30]. Similar results could be obtained when increasing the depth of the charge/discharge by utilizing 50-μm lithium foils to fabricate symmetrical lithium cells. The incorporation of 1,3-dithiane facilitated uniform lithium plating/stripping behavior, enabling long-term stability (for ≤500 h) at 1.0 mA cm^−2^ and 1.0 mAh cm^−2^ with an overpotential of ∼145 mV for the cell with LBE2S (Fig. S15a). In contrast, the cell with LBE exhibited a significant potential increase after 55 h under identical conditions. When the cycling conditions were further increased to 3.0 mA cm^−2^ and 3.0 mAh cm^−2^, the cell with LBE failed within 30 h due to a rapid rise in the overpotential (Fig. S15b). However, the cycling lifetime was extended to >300 h upon the addition of 1,3-dithiane. The results demonstrate that the carbonate electrolyte fails to generate a stable SEI on the lithium-metal surface, resulting in a rapid increase in the interface impedance, ultimately leading to cell failure. However, the improved cycle performance highlights the significant role of the 1,3-dithiane additive in enhancing interface stability.

To evaluate the effect of the 1,3-dithiane additive on electrode regulation, scanning electron microscopy was applied to observe the Li deposition with different electrolytes after 20 cycles. In LBE, the lithium deposition exhibits an uneven morphology, characterized by large cracks and fragmented areas (Fig. 3b). Upon further magnification, it becomes evident that the lithium deposition possesses a loose, porous structure (Fig. 3c) [31]. Cross-sectional analysis further corroborates this observation, revealing the broken porous deposits with uneven height and an average thickness of ∼126 μm (Fig. 3d). The uneven and highly porous Li deposition is believed to induce dendritic lithium formation and aggravate the active lithium loss due to continuous side reactions, ultimately leading to a rapid decline in the cell capacity [32,33]. In contrast, a much more uniform Li deposition morphology without any noticeable cracks can be obtained after the introduction of 1,3-dithiane additives (Fig. 3e–g). The presence of compact Li deposition confirms that 1,3-dithiane can effectively inhibit dendritic lithium formation, which is favorable for suppressing side reactions and stabilizing the electrode interface, and is conducive to improving the cycling lifespan and CE of LMBs [34]. Under the evaluated cycling conditions (3.0 mA cm^−2^, 3.0 mAh cm^−2^) (Fig. S16), the LBE electrolyte exhibited numerous porous fragments, indicating that the growth of deposited lithium was irregular and porous. In contrast, the LMA demonstrated a dense and dendrite-free morphology after cycling, which significantly enhances the cyclability of LMAs. Furthermore, the thickness of the corrosion layer and lithium-deposition layer formed on the surface of the anode using LBE was measured at 264.4 μm, which is considerably larger than the surface layer formed with LBE2S, which measured at only 147.1 μm. This finding confirms the beneficial role of the 1,3-dithiane additive in stabilizing the SEI and regulating lithium-deposition behavior.

Furthermore, electrochemical impedence spectra (EIS) measurements were conducted to investigate the electrode interface characteristics (Fig. S17). The corresponding equivalent circuit and the impedance parameters obtained from fitting are presented in Table S4. After 20 cycles, two semicircles representing the solid electrode interface (*R*_SEI_) and the resistance of charge transfer (*R*_ct_) of symmetrical cells with LBE2S are 32.11 and 11.42Ω, respectively, which are smaller than those obtained from the LBE electrolyte (73.75 and 17.16 Ω, respectively). The lower *R*_SEI_ indicates that the addition of the 1,3-dithiane additive could effectively improve lithium-cation transportation within the SEI and a low charge-transfer resistance (*R*_ct_) represents an accelerated electrochemical redox reaction at the interface, which is consistent with the remarkable cycling performance of symmetrical cells cycled at high current density. Moreover, the Tafel plot (Fig. S18) shows that the SEI formed in the LBE2S electrolyte presents an exchange current density of ∼2.14 mA cm^−2^, which is ∼2.4 times higher than that of the LBE electrolyte owing to the enhanced lithium-cation transport kinetics of the inorganic-rich SEI derived from the preferential reduction of 1,3-dithiane additives on the anode [35]. The lithium-ion-diffusion coefficient after 20 cycles was evaluated to assess the interface kinetics (Fig. S19). Benefitting from reduced interface impedance and energy barrier, the Li-ion-diffusion coefficient of the cycled Li-anode interface improves from 9.9 × 10^−10^ to 3.3 × 10^−9^ cm^2^ s^−1^ when applying the 1,3-dithiane additive, ensuring accelerated Li^+^ migration across the electrolyte/electrode interface. Afterwards, the activation energies of each interfacial process were determined according to the classic Arrhenius law based on the temperature-dependent EIS (Fig. 3h and i, and Fig. S20). In the LBE2S electrolyte, the activation energy for Li^+^ transport through SEI (*E*_a, SEI_ = 18.8 kJ mol^−1^) is remarkably lower than that in LBE (*E*_a, SEI_ = 85.4 kJ mol^−1^). A lower *E*_a, SEI_ signifies the rapid diffusion of Li ions through the SEI, allowing sufficient Li ions beneath the SEI to alleviate the formation of dendritic Li in LBE2S. Besides, a lower Li^+^ desolvation energy (29.7 kJ mol^−1^ for LBE2S and 59.5 kJ mol^−1^ for LBE) demonstrates the effectiveness of 1,3-dithiane at regulating the Li^+^ desolvation behavior near the lithium-metal anode [36,37].

The *in situ* optical lithium symmetrical cells were assembled to observe the lithium-growth process directly (Fig. 3j and k). After the 1,3-dithiane additive was put in, the loose and unconstrained lithium-deposition behavior was obviously alleviated, suggesting that 1,3-dithiane could promote lithium-deposition behavior even in the absence of external pressure.

### Enhanced electrode interphases

**Figure 4.**
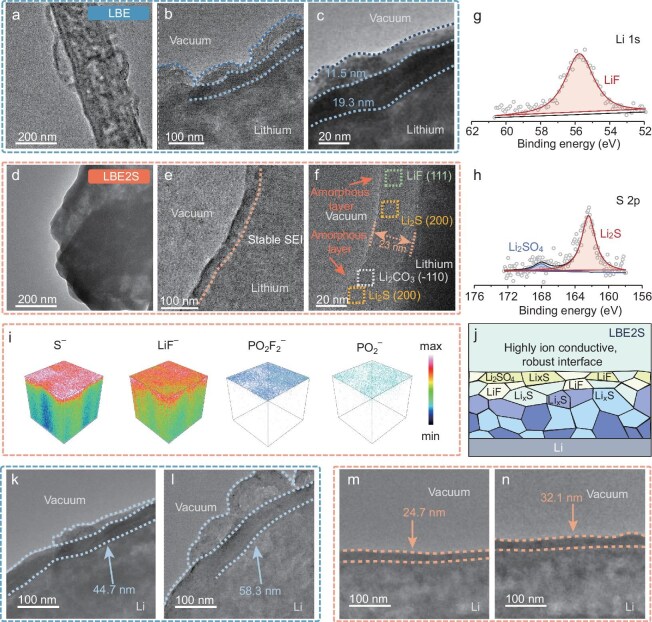
Electrode interface characterization. Cryo-TEM images of 0.3 mAh cm^−2^ deposited lithium metal using (a–c) LBE and (d–f) LBE2S. (g) Li 1s and (h) S 2p high-resolution XPS spectra for the anode cycled in LBE2S. (i) TOF-SIMS 3D reconstruction of the sputtered volume of several secondary ion fragments on anode. (j) Schematic diagram of SEI composition and structure. Cryo-TEM images of 0.1 mAh cm^−2^ deposited lithium metal using LBE for (k) 5 and (l) 10 cycles, and LBE2S for (m) 5 and (n) 10 cycles.

The nanoscale structure and composition of the SEI were further evaluated by using cryogenic transmission electron microscopy (cryo*-*TEM). As illustrated in Fig. 4a–f, the SEI formed in the blank electrolyte exhibits an uneven morphology characterized by a fluctuant outermost amorphous organic layer (Fig. 4a and b) [38]. Notably, no significant crystalline species were observed within the SEI, which has a total thickness of ∼30.8 nm (Fig. 4c). The presence of a thick and highly vulnerable SEI formed from carbonate electrolytes hinders the kinetics of interfacial ion conduction and leads to rapid consumption of both the electrolyte and the active lithium.

In comparison, the uniform SEI layer generated from the LBE2S electrolyte (Fig. 4d and e), measuring ∼23 nm in thickness (Fig. 4f), reveals a consistent structure in which inorganic nanocrystals are embedded within amorphous organic species. This is further covered by a thin amorphous surface layer of just a few nanometers [39]. The uniform and inorganic-rich SEI derived from LBE2S would significantly enhance lithium-ion diffusion and reduce resistance, promoting a more compact lithium deposition and mitigating side reactions [40]. Moreover, inverse fast Fourier transform analysis reveals that the magnified lattice spacing of 0.231, 0.334 and 0.419 nm corresponds well with the (111), (111) and (–110) crystal planes of LiF, Li_2_S and Li_2_CO_3_, respectively (Fig. S21). Consequently, the cryo-TEM studies demonstrate that, in conventional carbonate electrolytes, the lithium-metal surface tends to form a thick and uneven SEI. Conversely, in LBE2S electrolytes, the introduction of additives improves the local solvent environment at the interface, promoting anion participation in the film-formation process. Additionally, the reduction of 1,3-dithiane additives at the lithium-anode interface collaborates to foster the formation of an inorganic-rich SEI. This results in improved interface stability and enhanced ion conduction.

The surface chemistry of cycled lithium anodes derived from different electrolytes was subsequently probed by using X-ray photoelectron spectroscopy (XPS). The C 1s spectra in Figs S22a and S23a show similar components for the anodes cycled in LBE and LBE2S, while a higher proportion of ROCO_2_Li species in SEI could be observed for the control sample (Fig. S37), suggesting inferior SEI properties for the anode cycled in the LBE electrolyte due to the inadequate mechanical strength and poor lithium-cation conductive properties of the amorphous organic component [8,9]. The observation of fewer ROCO_2_Li species for the SEI formed in the LBE2S electrolyte also confirmed the effect of the additive on the elimination of organic compounds. In the Li 1s and F 1s spectra (Fig. 4g and Figs S22b and c and S23b), the peaks at about 55.2, 55.7, 685.0, 686.7 and 687.3 eV are attributed to RCOOLi, Li–F, LiF, Li*_x_*PF*_y_* and PO*_x_*F*_y_*, respectively [41,42]. Apparently, a higher proportion of LiF species (55.7 eV in Li 1s and 685 eV in F 1s) in the SEI can be observed (Fig. S38). Additionally, the clear difference in the S 2p spectra in Fig. 4h and Fig. S22d confirmed that 1,3-dithiane prefers to participate in forming a multiphase SEI in the presence of sulfur-rich products, including Li_2_SO_4_ (168.0 eV) and Li_2_S (163.7 eV) species. The quantified SEI composition ratio plots in Fig. S24 confirm that Li_2_S dominates the sulfur-containing species in the SEI. Among them, the ionic conductor of Li_2_S could decrease the lithium-cation transfer resistance and promote uniform lithium-deposition/stripping behavior [43].

Time-of-flight secondary ion mass spectrometry (TOF-SIMS) was conducted to further investigate the composition of the SEI formed in LBE2S (Fig. 4i and Figs S25 and S26). Sulfur-containing inorganic species and LiF were distributed throughout the SEI (represented by S^−^ and LiF^−^) and enriched in the top layer of the SEI. These findings suggest that 1,3-dithiane could participate in modifying the sulfur-rich SEI. Moreover, combined with the anion-decomposition products of PO_2_F_2_^−^ and POF_2_^−^ distributed in the SEI, robust and ionically conductive interphases can be formed on the anode, effectively enhancing the interface kinetics and long-term structural stability, leading to significantly improved cycling stability of the LMBs (Fig. 4j).

To further clarify the compositional evolution of the SEI layers, depth-dependent XPS analysis was conducted on lithium anodes after 100 cycles to probe the spatial distribution of the SEI components (Figs S27–S29). For the LBE-derived SEI, the C 1s spectra reveal a pronounced attenuation of organic characteristic peaks with increasing etching depth, indicating a double-layer structure in which organic species predominantly accumulate in the outer layer, while the inner layer still retains a significant proportion of organic components. This gradient structure is consistent with the mechanical instability of an organic-rich SEI, which readily fractures during cycling, exposing fresh Li surfaces to continuous parasitic reactions. In stark contrast, the LBE2S-derived SEI exhibits minimal variation in organic peak intensity across etching depths, confirming a homogeneous and inorganic-rich SEI with suppressed solvent decomposition. The Li 1s spectra further corroborate this distinction. The LBE-derived SEI shows a higher proportion of ROCO_2_Li in the outer layer, indicative of solvent-derived organic species. Conversely, the LBE2S-derived SEI is dominated by LiF throughout the entire depth, confirming that the 1,3-dithiane additive promotes preferential anion decomposition and suppresses solvent-derived organic accumulation. This aligns with the mechanistic role of 1,3-dithiane in enhancing anion participation in SEI formation via strong PF_6_^−^–additive interactions. The S 2p spectra highlight the direct contribution of the additive to SEI chemistry. The LBE2S-derived SEI features sulfur-rich species (Li_2_SO_4_ and Li_2_S), with S_2_^−^ species enriched in the inner SEI layer. This spatial distribution suggests that 1,3-dithiane not only participates in forming sulfur-containing inorganic components (Li_2_S and Li_2_SO_4_) at the interface, but also undergoes further reduction near the Li-metal surface, generating highly ionic conductive S_2_^−^-containing species in the inner SEI. The synergistic coexistence of LiF and Li_2_S within the SEI significantly enhances mechanical stability and interfacial kinetics. Consequently, the LBE2S-derived SEI exhibits a homogeneous, inorganic-rich architecture with minimal organic content, low Li^+^-diffusion resistance and superior electronic insulation. This unique SEI structure facilitates uniform Li deposition, mitigates dendrite growth and ensures long-term cycling stability.

We further conducted cryo-TEM analysis to unveil the structural evolution of the SEI along with cycles (Fig. 4k–n). The cryo-TEM results indicate that the SEI of lithium cycled in LBE undergoes a thickening process, with the inner SEI increasing in thickness from 44.7 to 58.3 nm. The outer SEI, however, exhibited uneven growth, with rapid thickening in localized areas suggesting significant side reactions occurring in those regions, which highlights the fragility and inhomogeneity of the SEI formed in LBE. In contrast, the compact SEI formed in LBE2S displayed a thin and uniform nanostructure, with a notably slower thickening rate, increasing from 24.7 to 32.1 nm. These results demonstrate that the inorganic-rich SEI guided by the 1,3-dithiane additive possesses good stability and uniformity.

### Electrochemical properties in full cells

**Figure 5.**
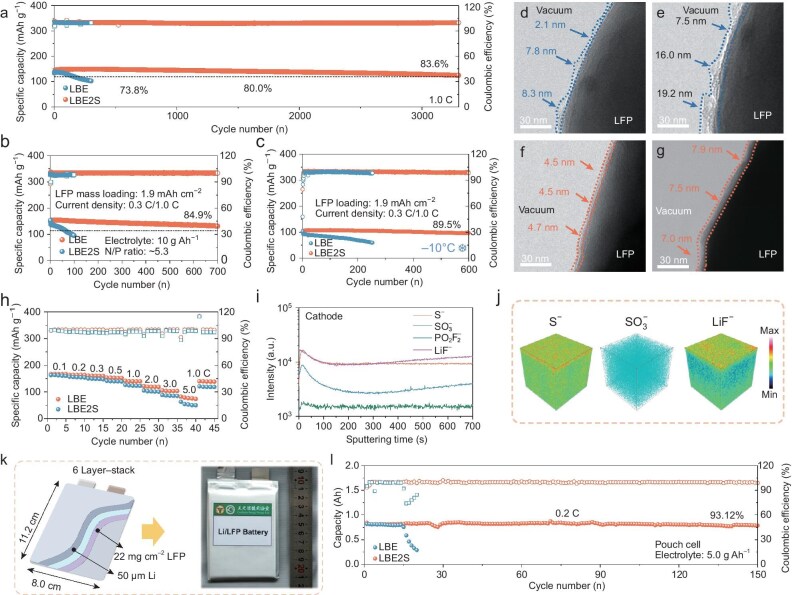
Electrochemical performance of Li//LFP full cells. (a) Galvanostatic cycling at 1.0 C. (b) Cycling performances of Li//LFP coin cells with ultra-thin lithium anode of 50 μm and a controlled electrolyte amount of 10 g Ah^−1^. (c) Cycling performances of Li//LFP coin cells at −10°C. TEM images of LFP cathode cycled in LBE for (d) 100 and (e) 200 cycles, and LBE2S for (f) 100 and (g) 200 cycles. (h) Rate performance of Li//LFP full cells. (i) Depth profiling of several secondary ion fragments on LFP cathode in LBE2S. (j) TOF-SIMS 3D reconstruction of the sputtered volume of several secondary ion fragments on cathode. (k) Diagram of the assembled pouch cell and corresponding digital image. (l) Long-term cycling performance of pouch cell with 1,3-dithiane additive-containing electrolyte.

To evaluate the reversibility of LFP full cells with the 1,3-dithiane electrolyte additive, cyclic voltammetry (CV) scans were conducted for the first three cycles (Fig. S30). Full cells with LBE and LBE2S electrolytes exhibit completely reversible redox peaks, indicating that the addition of 1,3-dithiane would not affect the normal operation of LFP cathodes. Notably, the cell with LBE2S shows smaller polarization of ∼600 mV than that of the control sample with LBE (660 mV) for the first cycle, implying fast lithium-cation migration and superior electrode interface properties in the LBE2S-based full cell [44]. In the subsequent two cycles, the LFP full cell with the LBE electrolyte maintains stable polarization, while decreased polarization observed in the full cell with LBE2S verifies the gradual formation of an electrode interface with enhanced lithium-cation transfer kinetics. The linear relationship observed between the square root of the sweep speed (*v*^1/2^) and the peak current (*I*_p_) illustrates that the lithium-cation insertion–extraction processes are dominated by diffusion behavior. According to the Randles–Sevcik equation, the diffusion coefficients of the lithium ions (*D*_Li^+^_) are directly proportional to the slope of the line relating *v*^1/2^ to *I*_p_. As shown in Fig. S31, the slope for the LBE2S-based cell is greater than that for the cell with LBE, suggesting that the *D*_Li^+^_ for the LBE2S-based cell is higher. This indicates that the incorporation of 1,3-dithiane into the electrolyte enhances the diffusion and transfer of lithium cations, thereby improving the high-rate performance of the LFP cathode. To systematically assess the superiority of the 1,3-dithiane additive, the galvanostatic intermittent titration technique (GITT) was implemented to analyse the diffusion kinetics of the lithium cations. Figure S32 presents the GITT curves for both electrolytes during the 101st charge/discharge cycle at a rate of 0.1 C. The results indicate that the LBE2S-based LFP full cell demonstrates a higher *D*_Li^+^_ compared with the cell with LBE, which aligns with the CV findings. It can be concluded that 1,3-dithiane not only facilitates the construction of a stable CEI, but also enhances rapid ion transport at the interface. This improvement is attributed to the formation of a coherent interface and a reduced energy barrier for lithium-cation migration channels. Overall, these findings suggest that 1,3-dithiane significantly improves lithium-cation insertion and extraction efficiency, thereby promoting more effective redox reactions in LFP cathodes. Afterward, the long-term cycling performance of LFP full cells cycled at 1.0 C with LBE2S was assessed to evaluate the superior compatibility of the LBE2S electrolyte, which shows a high initial CE of 96.3% and an average CE of >99.4% for 3300 cycles (Fig. 5a). Consequently, an improved discharge capacity-retention rate of 83.6% after 3300 cycles can be achieved. In contrast, the full cell with LBE electrolyte exhibits only 73.8% of the maximum discharge capacity after only 300 cycles. The remarkably enhanced cycling performance could be ascribed mainly to the inhibition of parasitic reactions by the 1,3-dithiane-derived CEI and SEI [31]. Consequently, the LBE2S electrolyte delivers a level of cyclability and efficiency that surpasses those of many established electrolyte regulation strategies, positioning it as a promising solution for practical LMBs (Table S5). The corresponding charge/discharge medium voltage (V vs. Li^+^/Li) profiles in Fig. S33 show that the polarization of the LFP full cell with LBE expands greatly with cycling and a high overpotential of ∼207.1 mV is obtained after 300 cycles. In contrast, the cells with the LBE2S electrolyte maintain stable and low polarization with a low overpotential of 90.9 mV at the 300th cycle. This difference confirms the formation of a stable, low-impedance interface on both the cathode and the anode enabled by the 1,3-dithiane additive [45,46].

Furthermore, under more realistic conditions for practical LMBs, LFP full cells were constructed by pairing a thin lithium-foil anode (50 μm thick, ∼10 mAh cm^−2^) and a high-areal-loading LFP cathode (∼1.9 mAh cm^−2^) with a controlled electrolyte-to-cathode ratio of ∼10 g Ah^−1^ in each cell. The full cell with LBE2S retains a cycling lifespan of 700 cycles for 84.9% capacity retention (Fig. 5b), whereas the cell with LBE suffers from rapid capacity fading, with 80% capacity retention after only 70 cycles under these harsh testing conditions. The cycling performance of LFP full cells deteriorates noticeably with a decreased N/P ratio. For an N/P ratio of 3.16, LFP full cells with the LBE electrolyte experienced capacity decline after 70 cycles, accompanied by significant fluctuations in the CE (Fig. S34a). After 100 cycles, the cycling decline behavior intensified due to the rapid, irreversible loss of active lithium and gradual electrolyte depletion, stemming from severe side reactions at the lithium-anode interface. However, with the introduction of the additive, the capacity decline was alleviated, achieving a capacity-retention rate of 92.4% after 250 cycles. Similar trends were observed when the N/P ratio was further reduced to 1.05 (Fig. S34b). The combination of uneven and porous electrodeposited lithium with the reduced lithium excess exacerbated the performance degradation of the LFP full cells. Without the additive, the LFP full cells demonstrated significant capacity decline after ∼30 cycles, whereas a high capacity-retention ratio of 90.18% was achieved after 150 cycles when the 1,3-dithiane additive was introduced. These observations underscore the beneficial effect of the 1,3-dithiane additive in improving the stability of LMAs under harsh conditions, particularly in full cells with low N/P ratios. Figure 5c demonstrates the cycling performances of LFP full cells cycled at −10°C. The full cell with LBE2S exhibits exceptional low-temperature performance, retaining 89.5 % of the discharge capacity after 600 cycles. In comparison, the LFP full cell with LBE initially shows lower capacity than that with LBE2S, with a rapid decline in performance, leading to a capacity retention of only 79.9% after 100 cycles.

Meanwhile, the structure and components of the CEI on the LFP were also characterized (Fig. 5d–g). The uneven CEI on the LFP cycled in LBE shows a significant increase in thickness after 200 cycles, with the average thickness increasing from 6.1 to 14.2 nm. This observation suggests that the CEI undergoes severe processes of accumulation, dissolution and degradation. Such a CEI structure aggravates electrolyte penetration and reconstruction of the CEI, leading to continuous side reactions and rapid capacity decay. In contrast, the CEI of LFP cycled in LBE2S maintains its structural integrity, exhibiting a thin and uniform CEI that seamlessly wraps around the LFP. After 200 cycles, the thickness and uniformity of the CEI only undergo alleviated changes, with the average thickness increasing from 4.6 to 7.5 nm.

The rate performance of LFP full cells with different electrolytes was also investigated (Fig. 5h). Compared with the control sample, the LBE2S-based full cell sustains higher specific capacities, especially at high rates, which could be attributed to the efficient ion-transport kinetics enabled by the inorganic-rich electrode interface (CEI/SEI) derived from LBE2S. These results demonstrate the superior compatibility of 1,3-dithiane with both the LFP cathode and the lithium-metal anode; therefore, the reversible lithium-deposition/stripping behavior and stable LFP cathode interface can be enabled by the as-formed inorganic-rich SEI and CEI in LBE2S, which exhibit the great potential of the 1,3-dithiane additive for application in LMBs with a long cycle life.

The chemical compositions of the 1,3-dithiane-derived CEI on the LFP cathode were further revealed. The chemical composition of the surface of the cycled LFP cathodes derived from different electrolytes was subsequently probed by using XPS. In the C 1s spectra (Figs S35a and S36a), the main peaks at 284.80, 286.65, 288.85 and 290.20 eV are attributed to C–C, C–O, ROCO_2_Li and C–F, respectively [23,47]. The peak representing ROCO_2_Li could be mainly attributed to the decomposition products of the carbonate solvents during high-voltage operation. The cycled LFP in LBE2S shows a much weaker peak of ROCO_2_Li, suggesting less continuous electrolyte degradation and an enhanced CEI on the LFP (Fig. S37) [47]. Similar results were obtained from the F 1s and Li 1s spectra. For the F 1s spectra (Figs S35b and S36b), the reductive decompositions of PF_6_^−^ are detected at 684.8, 685.6, 686.8 and 687.0 eV, which are assigned to LiF, LiO*_x_*F*_y_*, Li*_x_*PF*_y_* and Li*_x_*PO*_y_*F*_z_*, respectively [12,22,23,42]. The content of LiF in the cathodes cycled in LBE2S is higher than that in LBE (Fig. S38), which signifies that 1,3-dithiane may accelerate the conversion of F-containing species into LiF. Besides, the Li 1s spectra in Figs S35c and S36c provide strong evidence that LiF (55.6 eV) is the dominant component for the cathodes cycled in LBE2S [41]. Moreover, the XPS spectra for S 2p (Fig. S35d) show that no sulfur-containing compounds were generated on the LFP cathode cycled in LBE. Conversely, promoted by 1,3-dithiane, typical characteristic peaks of C–S (163.5 eV) and Li_2_SO_3_ (165.6 eV) appear on the LFP cathodes cycled in LBE2S (Fig. S36d) [15,24], indicating the promotion of lithium-cation transport kinetics at the cathode interface by Li_2_SO_3_, which acts as a good lithium-cation conductor [45,48].

Besides, TOF-SIMS was conducted to further investigate the composition of the CEI in LBE2S. In Fig. 5i and j and Fig. S39, sulfur-containing species represented by S^−^ and SO_3_^−^ are uniformly distributed throughout the CEI, indicating the involvement of 1,3-dithiane additives in the formation of the CEI. Anionic decomposition products, including PO_2_F_2_^−^ and LiF_2_^−^, are also detected. The strong signals suggest the abundant fluorine-containing and sulfur-containing inorganic components within the CEI, which contribute to a robust and highly ion-conductive interlayer, effectively enhancing positive electrode interface kinetics (Fig. S40) [31].

To further illustrate the advantages of LBE2S in practical applications, 1-Ah-level pouch cells were assembled (Fig. 5k). The pouch cell with LBE exhibited an initial discharge capacity of 818.4 mAh and underwent sharp capacity fading after only 15 cycles (Fig. 5l). In contrast, a significantly prolonged cycling lifespan with a capacity retention of 93.1% after 150 cycles was obtained with the 1,3-dithiane additive. EIS analysis of the pouch cells after 10 cycles (Fig. S41 and Table S6) reveals that the cell with LBE2S exhibits an extremely small *R*_ct_ of ∼0.12 Ω, which is ∼20 times smaller than that of the cell with LBE (2.39 Ω) due to the lower resistance of charge transfer at the interface between the electrolyte and the electrode, which could mainly be ascribed to the facilitated lithium-cation transfer kinetics originating from the preferential decomposition of 1,3-dithiane on the electrode to form an inorganic-rich, highly conductive interface. In the corresponding charging/discharging voltage profiles of selected cycles (Fig. S42), both cells display a similar voltage polarization of ∼100 mV at the first cycle. However, a rapid increase in voltage polarization can be observed for the pouch cell with LBE after 20 cycles, indicating insufficient lithium-cation transfer kinetics on the electrode interface, which is associated with the accumulation of dead lithium and rapid consumption of electrolyte due to the unstable SEI formed in the carbonated electrolyte. In stark contrast, the cell with LBE2S presents an alleviated increase in voltage polarization of 400 mV after 80 cycles. Subsequently, the pouch cells, after 10 cycles, were disassembled in an argon-filled glovebox (Fig. S43). The lithium anode cycled in LBE was covered in a gray dead lithium layer and the electrolyte dried up completely. Moreover, some areas even show exposed copper current collectors, indicating severe lithium pulverization and complete consumption of the lithium metal. Due to the addition of 1,3-dithiane that could alleviate the lithium-pulverization behavior, a dark-gray decomposition products covering the fresh lithium can be found. Additionally, the LFP cathode shows no significant structural damage and remains wet. These results further endorse the fact that the 1,3-dithiane additive could enhance the electrode interface and mitigate active lithium loss to prolong the cycling lifespan of LMBs through the formation of a rich inorganic interface.

The cycling performance demonstrates that the Li//LiNi_0.8_Co_0.1_Mn_0.1_O_2_ (NCM811) full cell with the LBE2S additive retains 87.37% of its initial capacity after 100 cycles at 1 C, significantly outperforming the baseline electrolyte, which exhibits a specific capacity retention of 70.34% after 100 cycles under identical conditions (Fig. S44a). The corresponding charge–discharge profiles indicate that the cell with the LBE2S electrolyte consistently maintained a higher discharge-specific capacity and lower polarization throughout the testing period (Fig. S44b and c). This improvement highlights the enhanced compatibility of LBE2S with high-voltage NCM cathodes compared with conventional carbonate-based electrolytes. Further mechanistic insights were obtained through TEM analysis of the cycled NCM811 cathodes. As shown in Fig. S45, the LBE2S-containing electrolyte promotes the formation of a uniform, compact CEI layer with an average thickness of ∼8.3 nm, which effectively mitigates electrolyte decomposition and parasitic reactions. In contrast, the LBE generates a heterogeneous and thicker CEI layer (12.3 nm) with visible inhomogeneity, exacerbating interfacial instability and structural degradation.

## CONCLUSIONS

In summary, we have investigated a high-sulfur-content (53.5 wt%) electrolyte additive, 1,3-dithiane, for ultra-stable LMBs. Theoretical calculations and electrochemical analyses have verified that the 1,3-dithiane additive preferentially decomposes at both the anode and the cathode interfaces, contributing a robust and highly ion-conductive interface. Meanwhile, the strong interaction between 1,3-dithiane adsorbed on the lithium-metal anode and PF_6_⁻ leads to the decomposition of PF_6_⁻, facilitating the formation of LiF at the electrode interface. Additionally, 1,3-dithiane reacts with strong bases, such as alkyl lithium, to form 2-lithium-1,3-dithiane. The reaction effectively inhibits the further generation of alkyl lithium, preventing the formation of organic components within the SEI. Simultaneously, 2-lithium-1,3-dithiane decomposes on the lithium-metal surface, resulting in the creation of a sulfur-rich SEI, which transforms undesirable organic components into beneficial sulfur-based constituents. Moreover, as a polarity-reversal component, 2-lithium-1,3-dithiane enhances the resistance of carbonate solvents to nucleophilic attack, reduces solvent decomposition at the interface and minimizes the formation of organic components within the SEI. As a result, on the anode side, an SEI enriched with Li_2_S and LiF could facilitate the lithium-cation transfer kinetics and the stability of the SEI, leading to a four-times cycling-lifespan improvement in lithium symmetrical cells for compact and uniform lithium deposition without obvious dendritic lithium. Regarding the cathode, superior structural stability and a higher lithium-cation-conductive interface is enabled by a CEI incorporating Li_2_SO_3_, C–S species and LiF. A synergistic enhancement of both the cathode and the anode interfaces effectively suppresses active lithium loss due to undesirable electrode/electrolyte interactions. Therefore, a Li//LFP full cell with a 2.0-wt% 1,3-dithiane-containing electrolyte exhibits a high capacity retention of 83.6% after 3300 cycles at 1.0 C. More importantly, an Ah-level Li//LFP pouch cell exhibited a >10-times increase in the cycling lifespan with a 93.1% capacity retention after 150 cycles. This work offers a simple and effective strategy to develop advanced electrolytes to boost the cycling performance of LMBs.

## Supplementary Material

nwaf259_Supplemental_File
