# Supplementary material for: A thioether additive as an interfacial regulator for ultra-stable lithium-metal batteries
Source: Natl Sci Rev. 2025 Jun 30;12(8):nwaf259. doi: 10.1093/nsr/nwaf259 (PMC12374724; doi:10.1093/nsr/nwaf259)
Supplement: nwaf259_Supplemental_File [file nwaf259_supplemental_file.pdf]

Supplementary Information
A thioether additive as interfacial regulator for ultra-stable lithium metal batteries

Xiaosong Xiong[1], Shuanglong Xu[1], Wenjie Zhang[1], Qiao Qiao[2], Yuan Ma[1], Yiren Zhong[1], Xin-Bing Cheng[1], Jiarui He[1], Zhi Zhu[1], Faxing Wang[1], Tao Wang[1]*, Yuping Wu[1]*

[1] Confucius Energy Storage Lab, School of Energy and Environment & Z Energy Storage Center, Southeast University, Nanjing 211189, China
[2] School of Chemistry and Molecular Engineering, Nanjing Tech University, Nanjing 211816, China

*Correspondence to: wangtao2021@seu.edu.cn; wuyp@seu.edu.cn

METHODS

Material preparation.

1,3-dithiane was purchased from Aladdin and used without further purification. The baseline electrolyte, composed of 1 mol L^{-1} $LiPF_6$ dissolved in EC: DMC: EMC (1:1:1 by weight ratio), was provided by Zhangjiagang Guotai-Huarong New Chemical Materials Co., Ltd. and labelled as LBE. The modified electrolyte, labelled as LBE2S, was prepared by dissolving 2.0 wt.% 1,3-dithiane in LBE. Lithium metal foil with a thickness of 500 μm and 15.6 mm in diameter was purchased from Canrd Technology Co., Ltd. 50 μm thin lithium anode was prepared by punching the lithium/copper composite strip (50 μm Li and 11 μm Cu, Canrd Technology Co. Ltd.) into disks with a diameter of 12 mm. For the 50 μm thin lithium electrode used in pouch cells, two lithium foils (7.0 × 8.4 cm^2) were rolled onto both sides of a copper electrode to construct a double-layer Li anode. The LFP and NCM811 cathode was prepared by mixing LFP or NCM811 (80 wt.%), acetylene black (10 wt.%), and poly(vinylidene fluoride) (PVDF) (10 wt.%) in N-methyl-2-pyrrolidone solvent (99.9%, electronic grade, Aladdin) to form uniform slurry via all-round planetary mechanochemical ball milling at 300 rpm for 2 h (LGB04, Nanjing Bo yun tong Instrument Technology Co., Ltd.). The slurry was then cast on aluminum foil by using a doctor blade, followed by drying in oven at 80 °C for 3 h and in vacuum oven at 120 °C for overnight. The typical active material loading of the resulting LFP electrodes was about 2.4 mg cm^{-2} and 12.0 mg cm^{-2}, respectively. Besides, the active material loading of the resulting NCM811 electrodes was about 9.8 mg cm^{-2}. Double-layer LFP active materials were cast on carbon-coated aluminum foil and die-cut into cathodes (6.7 × 8.0 cm^2) after thorough drying. The active material mass loading for the corresponding cathode was controlled as about 22.0 mg cm^{-2}. The separator used was DKJ-25 (25 μm thick, porous polypropylene with 45% porosity), provided by Zhejiang Dikunjian New Energy Technology Co., Ltd.

Material characterization.

Nuclear magnetic resonance (NMR) spectra of the electrolyte were obtained using acetonitrile-d3 as solvent in a liquid NMR (Bruker AV-III 600 MHz). Confocal

Raman spectra of electrolytes were recorded on a WITec alpha300 R instrument (excitation wavelength = 532 nm, acquisition time = 10s, accumulations = 10, filter = 1%, spectral region = 400~2000 cm^{-1}) using a custom mold with a quartz plate to isolate the air. The contact angle analysis was carried out using an optical contact angle meter (SL200KS, Kino, USA), and 3.0 μL droplet of the LBE and LBE2S electrolyte were typically used in the experiment. Scanning electron microscopy (SEM) and energy dispersive spectrometer (EDS) images were obtained by Zeiss Merlin Sigma 360 scanning electron microscope at an acceleration voltage of 15 kV. For observing the morphology of lithium deposition, lithium anode samples were collected by disassembling Li//Li cells after specific cycles in an argon-filled glovebox, followed by a gentle rinse with EMC solvent. After drying thoroughly, the lithium foils were sealed in an argon-filled plastic bag and quickly transferred to the SEM. To assess the interphase composition of cycled LFP cathode and lithium anode, the full cells underwent specific cycles were disassembled in an argon-filled glovebox. The cathode and anode samples were collected and gently rinsed with EMC solvent. After thorough drying, the samples were stored in sealed vials and transferred to another argon-filled glovebox to complete the sample preparation. Next, the samples were transferred to the XPS and TOF-SIMS via a customized vacuum chamber to ensure no exposure to air. X-ray photoelectron spectroscopy (XPS) was performed using a K-Alpha+ photoelectron spectrometer (Thermo Scientific) with Al Kα radiation. XPS depth profiles were obtained through Ar^+ sputtering at 3 keV for varying durations of 0, 50, 100, and 200 seconds. Time-of-flight secondary ion mass spectrometry (TOF-SIMS) analysis was carried out using a ToF-SIMS 5-100 instrument from IONTOF GmbH, the ion species is Bi_3^+ and raster size is 50 × 50 $μm^2$. To prepared the LFP and NCM811 samples for TEM characterization, the cycled LFP and NCM811 cathodes were soaked in DMC solvents and ultrasonic for about 10 min. Afterwards, 1-2 droplets of the uniformly dispersed suspension was dropped onto a Cu grid in a glove box. After thorough drying, TEM characterization was performed on FEI Tecnai G2 20. The morphology of SEI was examined using a Themis 300-300KV transmission electron microscope equipped with a cryo-transfer

station. Lithium metal was deposited onto a Cu grid with a capacity of 0.3 mAh cm^{-2} deposited at 0.1 mA cm^{-2} in Li//Cu cells. Subsequently, the Cu grid was immersed in DMC to eliminate any residual lithium salts and dried before freezing. For the cryo-TEM characterization, Cu grid was directly mounted to the holder and maintained at a low temperature of −170 °C. In situ optical observation of Li deposition in LBE and LBE2S were carried out using an optical microscope (YD650, Yuescope, China). Li//Li symmetrical cells were assembled in a homemade mold with two identical 10 mm square lithium foil (thickness ~500 μm). To ensure the air-tightness of the optical mold cell, a lithium foil was sealed within the cell and left exposed to air for one day. The maintenance of the metallic luster of lithium confirmed the integrity of the optical mold cell. Subsequently, sufficient electrolyte was added to eliminate any bubbles. During the testing period, a constant current discharge process was carried out at a current density of 1.0 mA cm^{-2} for 1 hour. The ionic conductivity of the electrolyte was directly measured using an ionic conductivity meter (Inesa Scientific Instrument Co., Ltd., DDBJ-351L) in a glove box at a temperature of 28 degrees Celsius.

Electrochemical measurement.

Based on CR2025 and CR2032 configuration (Shenzhen Kejing Star Technology Co., Ltd), all coin cells were assembled in an argon-filled glove box with H_2O and O_2 levels below 1.0 ppm (VG1200/750TS, Vigor Technologies (Suzhou) Co., Ltd.). Typically, 50 μL LBE or LBE2S electrolyte was employed in each coin cell unless specified otherwise. The galvanostatic charge/discharge measurements of coin cells were carried out using a NEWARE battery test system (CT-4008T, Shenzhen, China) at 25 °C. Li//Cu half cells were constructed with Cu foils (16 mm) as cathode and Li foils (12 mm) as anode. The CE evaluation of various 1,3-dithiane concentration involved an initial 5.0 h discharge and subsequent charging to 0.5 V to establish the SEI on the Cu electrode. Afterwards, the cells underwent 10 cycles of 1.0 h charge and discharge process after a 5.0 h discharge period, concluding with a final charge to 0.5 V. The entire procedure was performed at a current density of 1.0 mA cm^{-2}. Two identical Li foils (500 μm/50 μm) were assembled into Li//Li symmetric cells based on the CR2032 configuration. For long-term cycling stability evaluation, a current

density of 1.0 mA cm^{-2} or 3.0 mA cm^{-2} with a fixed charge/discharge time of 1 h was set. Besides, the rate performance of symmetric cells was cycled for 5 cycles at the current densities of 0.1, 0.2, 0.3, 0.5, 1.0, 2.0, 3.0, 5.0, 3.0, 2.0, and 1.0 mA cm^{-2}, respectively, with a fixed discharge/charge time of 1.0 h. For LFP full cells, $LiFePO_4$ cathodes (LFP, 2.4 mg cm^{-2}) and 500 μm Li metal anodes were employed as the cathode and anode, respectively, the cells were cycled at a rate of 1.0 C (170 mA g^{-1}) between 2.5 V and 3.8 V for galvanostatic measurements. 12.0 mg cm^{-2} LFP cathodes and 500 μm Li metal anodes were applied for LFP cells when measuring rate performance. Specifically, the full cells were charged at 0.3 C to the cut-off voltage of 3.8 V, followed by discharging at 1.0 C to 2.5 V when 12.0 mg cm^{-2} LFP cathodes and 50 μm Li metal anodes were applied, the electrolyte was controlled as 10 g Ah^{-1}. The lithium metal electrodes with limited lithium source were prepared by disassembling half cells in the glove box after plating fixed amount lithium (2.0 mAh cm^{-2}) on bare Cu at 1.0 mA cm^{-2}. In GITT measurement, the LFP full cells after 100 cycles were charged up to 3.8 V at 0.1C for 20 min and then were left to rest for 60 min to attain equilibrium voltage. For NCM811 full cells, the

The diffusion coefficient (D_{Li+}) can be determined by the following relation:

$$D_{Li^+} = \frac{4}{\pi \cdot \tau} \cdot \left(\frac{m_B \cdot V_m}{M_B \cdot A}\right)^2 \cdot \left(\frac{\Delta E_S}{\Delta E_\tau}\right)^2 \quad (1)$$

where τ stands for constant current pulse duration of the GITT measurement, M_B, V_m, m_B, and A represent the molecular weight, molar volume, electroactive mass, and surface area of the electrode, respectively. While the ΔE_S and ΔE_τ mean the change of steady-state voltage and a cell voltage variation at the corresponding (de) lithiation step, which can be acquired from the single-step GITT titration.

The Li ion diffusion coefficient was calculated by the formula as following:

$$D = \frac{R^2 \cdot T^2}{2 \cdot A^2 \cdot n^4 \cdot F^4 \cdot C^2 \cdot \sigma^2} \quad (2)$$

where R represents molar gas constant (8.314 J K^{-1} mol^{-1}), T presents room temperature (298.15 K), A presents the area of electrode, n presents electron transfer number, F presents Faraday constant (96 485 C mol^{-1}), C presents the Li ions

concentration (1.0 mol L^{-1}), and σ was the slope of the fitting line obtained from Nyquist plots.

Chronoamperometry, linear sweep voltammetry (LSV), cyclic voltammetry (CV), Tafel plots, and electrochemical impedance spectroscopy (EIS) measurements were carried out on an electrochemistry station (CHI 660E, CH Instruments Ins.). Chronoamperometry was employed to measure the initial current (I_0) and steady current (I_s) of Li||Li cells with different electrolytes under a constant voltage bias of 10 mV (ΔV). The initial resistance (R_0) and the steady state resistance (R_S) were recorded for the Li^+ transference number calculation based on the equation below:

$$t_{Li^+} = \frac{I_S(\Delta V - I_0 R_0)}{I_0(\Delta V - I_S R_S)} \quad (3)$$

LSV was measured between 0–6 V at 1.0 mV s^{-1} based on Li//stainless steel (SS) coin cell configuration. Cyclic voltammetry curves of LFP full cells were conducted between 2.5 to 4.2 V with a scan rate of 0.5 mV s^{-1}. The Tafel plots were obtained by scanning the Li//Li symmetric cells at 1.0 mV s^{-1} from −0.2 to 0.2 V. Electrochemical impedance spectroscopy measurements were performed at frequencies ranging from 100 kHz to 0.1 Hz with an amplitude of 5 mV. Li//LFP pouch cells were assembled in an argon-filled glove box with H_2O and O_2 levels below 1.0 ppm (customization, Vigor Technologies (Suzhou) Co., Ltd), the LFP cathodes (6.7 × 8.0 cm^2) and Li anodes (7.0 × 8.4 cm^2) were stacked together using a Z-folding method. Aluminum and nickel tabs were welded to the cathodes and anodes via ultrasonic spot welding (MRX-DH800, Shenzhen Mingruixiang Automation Equipment Co., Ltd.), respectively. After packaging in an aluminum-plastic film bag, the electrolyte (5 g Ah^{-1}) was injected, and the pouch cell was vacuum-sealed to complete the construction process. (MRX-YF200, Shenzhen Mingruixiang Automation Equipment Co., Ltd.). The cycling measurements of pouch cells were carried out using a NEWARE battery test system (CT-4008T TWS, Shenzhen, China) at 25 °C between 2.5–3.8 V. LFP pouch cells were charged to 3.8 V at 0.2 C and then held at that voltage until the current dropped below 0.05 C, Subsequently, the cells were discharged to 2.5 V at 0.2 C.

Computational details.

The electrostatic potential (ESP) mapping was acquired by DFT calculations via the Gaussian 09 package, and the geometric structures and corresponding frequency optimizations were calculated with the B3LYP with the 6-311++G(d). The theoretical calculations of the lowest unoccupied molecular orbital (LUMO), highest occupied molecular orbital (HOMO), Gibbs free energy, Fukui functions, atom charge and adsorption energies were conducted using the DMol³ module within Materials Studios 2020. Density functional theory (DFT) was employed to conduct a comprehensive analysis of the electronic structures of 1,3-dithiane, DMC, EC, EMC, and $LiPF_6$. Initially, geometric optimization was undertaken to ascertain the most energetically favorable configurations of these substances in their stable states. Conforming to established literature, the present study employed the local density approximation (LDA) during the optimization process. Geometry optimization during computation was executed utilizing the Perdew-Wang-Ceperley (PWC) correlation exchange functional in conjunction with the DNP3.5 basis set. Convergence criteria for energy, force, and displacement were set at 1×10^{-5} Ha, 0.002 Ha $Å^{-1}$, and 0.005 Å, respectively. After geometric optimization, LUMO, HOMO energies and Gibbs free energy were computed for each compound. Furthermore, the Fukui function was evaluated to discern alterations in electron density during chemical transformations. The entire computational procedure rigorously adhered to quantum mechanical principles and methodologies, ensuring the precision and validity of the outcomes. For the treatment of the exchange-correlation functional, the Generalized Gradient Approximation (GGA) approach and Perdew-Burke-Ernzerhof (PBE) functional were employed to ensure the accuracy of our calculations. The Brillouin zone integrations used Monkhorst-Pack grids of 5 × 5 × 1 mesh. The basis set chosen for the calculations was the double numerical plus polarization (DNP) function. The self-consistent field (SCF) convergence criterion was stringently set to 2.0×10^{-5}Ha, ensuring a maximum force convergence of 4.0×10^{-3} Ha $Å^{-1}$ and a maximum displacement convergence of 5.0×10^{-3} Ha. Additionally, the SCF convergence threshold of 1.0×10^{-5} eV/atom was imposed. All calculations were conducted within

the 14.04 × 14.04 × 23.51 Å³ supercell consisting of 48 Li atoms. The Li-(100) slab consists of 4 layers and an xy-plane of dimensions 14.04 Å × 14.04 Å with 20 Å of vacuum in the z-direction. In order to calculate the adsorption energies (EAdsorption) of DMC, EC, EMC and Dithiane on Li-(100) slab, we use the following Equation (4).

$$\mathrm{E}_{Adsorption} = \mathrm{E}_{Total} - \mathrm{E}_{Slab} - \mathrm{E}_{X} \quad (4)$$

whereE_{Total}, E_{Slab} and E_{X}represents the total energy of the system, energy of Li-(100) slab, and energy of single anion or single solvent molecule (DMC, EC, EMC or 1,4-dithiane.

This work also searches the energy barriers associated with the dissociation reactions of Li_2CO_3 and $LiPF_6$ at the lithium layer. The search protocol implemented was complete LST/QST, and to ensure the accuracy of the results, the higher precision PBE functional was chosen for the calculations. The spatial feasibility of the migration pathway was assessed by calculating the particle distances for the intermediate hundred frames during the migration process. In this calculation process, the basis set chosen for the calculations was the double numerical plus polarization (DNP) function. The self-consistent field (SCF) convergence criterion was stringently set to 2.0 $\times 10^{-5}$ Ha, ensuring a maximum force convergence of 4.0×10^{-3} Ha $Å^{-1}$ and a maximum displacement convergence of 5.0×10^{-3} Ha.

Supplementary Figures

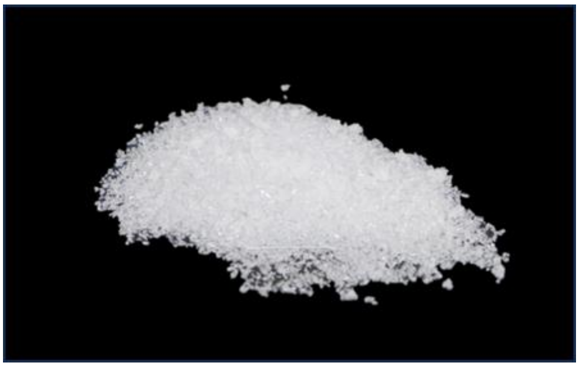

Figure S1. Digital image of the purchased 1,3-dithiane powder.

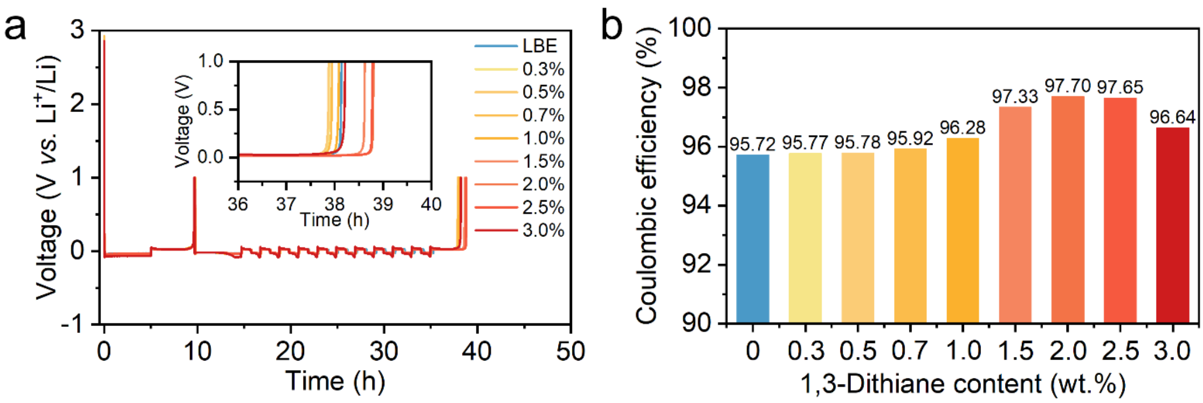

Figure S2. Comparison of the Coulombic efficiency of the LBE electrolyte with varying concentrations of 1,3-dithiane. (a) Voltage-time curves for Li//Cu half-cells, and (b) the corresponding calculated values of Coulombic efficiency.

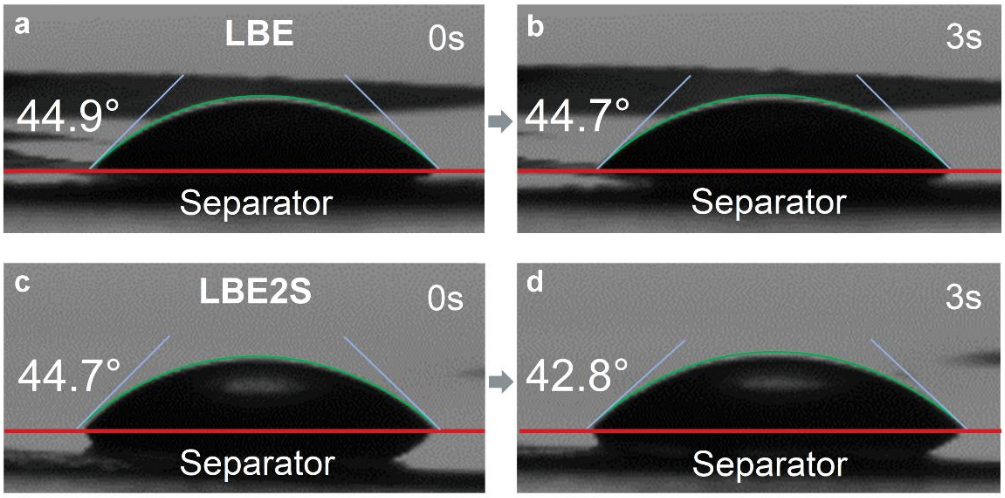

Figure S3. Contact angle of electrolyte on separator. (a) LBE on separator at 0 s and (b) after 3 s. (c) LBE2S on separator at 0 s and (d) after 3 s.

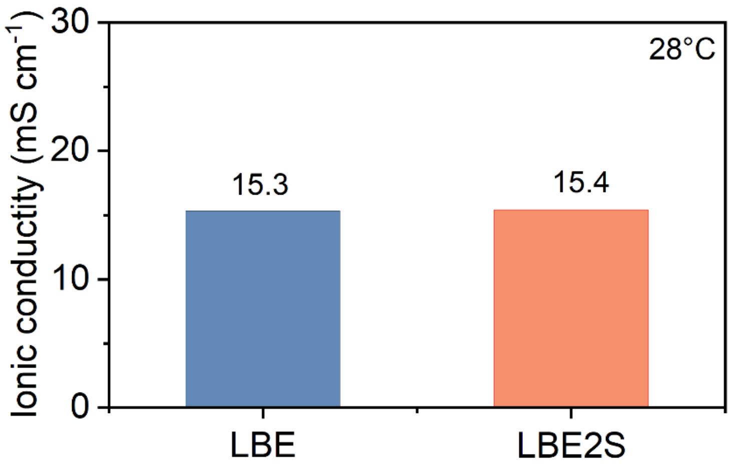

Figure S4. Ionic conductivity of LBE and LBE2S at 28°C.

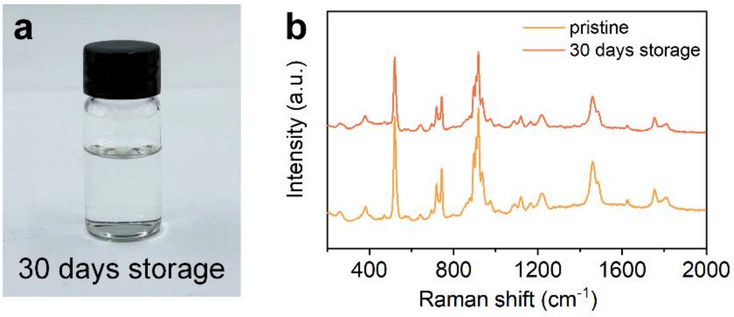

Figure S5. Long-term storage stability test. (a) Digital image of LBE2S after 30 days of storage. (b) Raman spectra of pristine LBE2S and LBE2S after 30 days of storage.

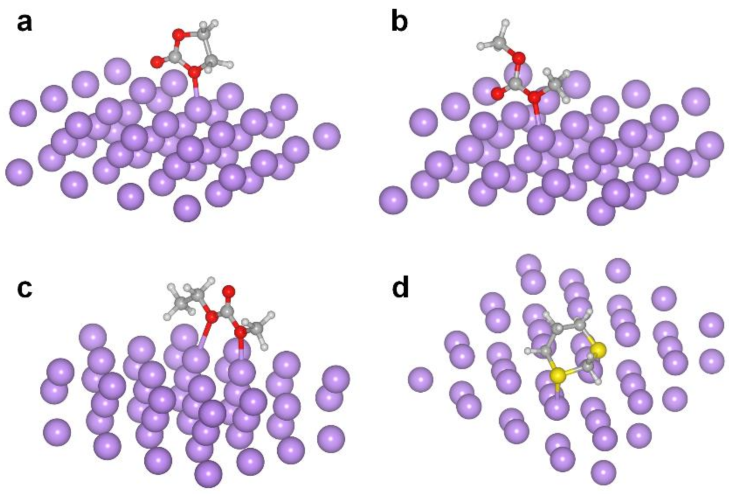

Figure S6. Adsorption energies of representative solvents (a) EC, (b) DMC, (c) EMC and (d) 1,3-dithiane on Li.

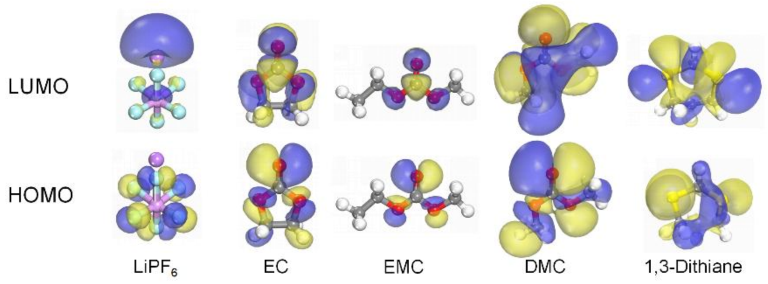

Figure S7. Electrolyte solvents and solutes molecular orbits.

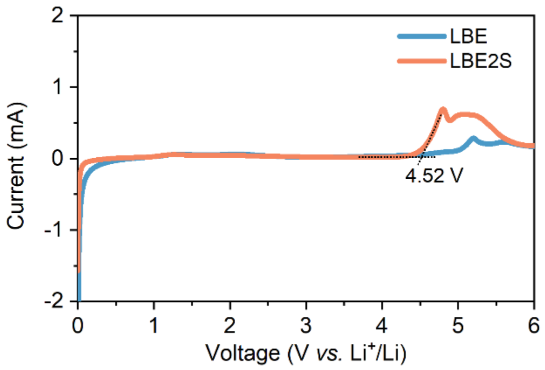

Figure S8. LSV curve of LBE and LBE2S.

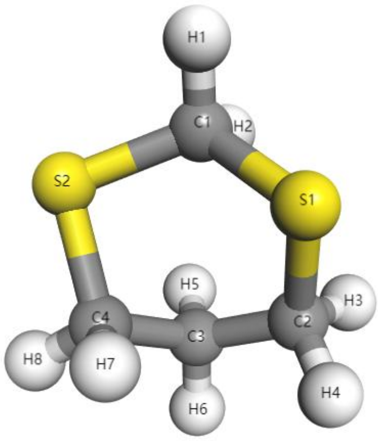

Figure S9. Ball-and-stick model of 1,3-dithiane after geometry optimization.

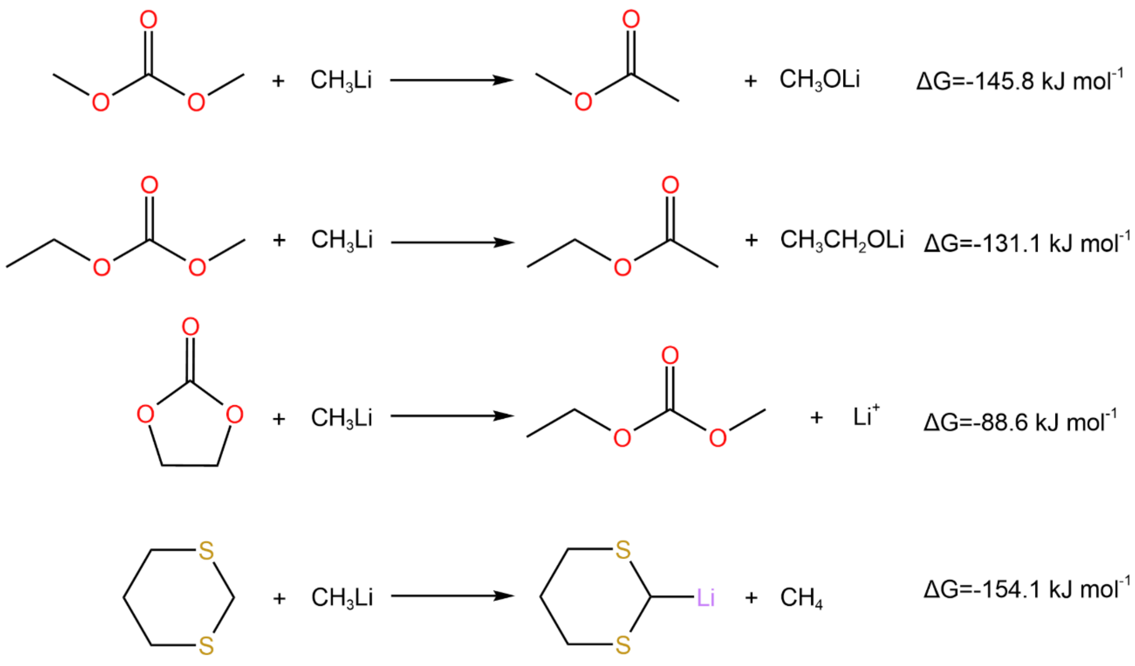

Figure S10. Reaction equation and Gibbs free energy of methyl lithium with carbonate solvent and 1,3-dithiane additive.

	2-lithium-1,3-dithiane
LUMO	-0.63 eV
HOMO	-4.18 eV

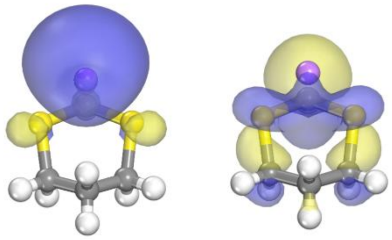

Figure S11. LUMO and HOMO energy level of 2-lithium-1,3-dithiane and corresponding molecular orbits.

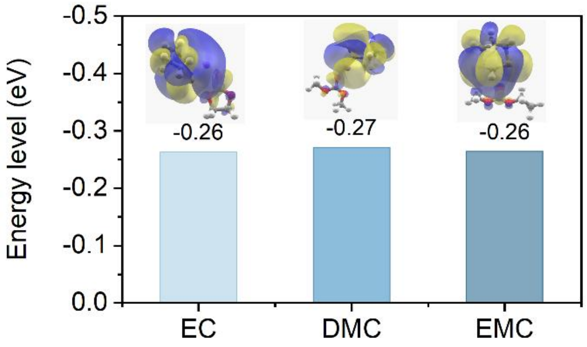

Figure S12. LUMO energy level of electrolyte solvents with 2-lithium-1,3-dithiane.

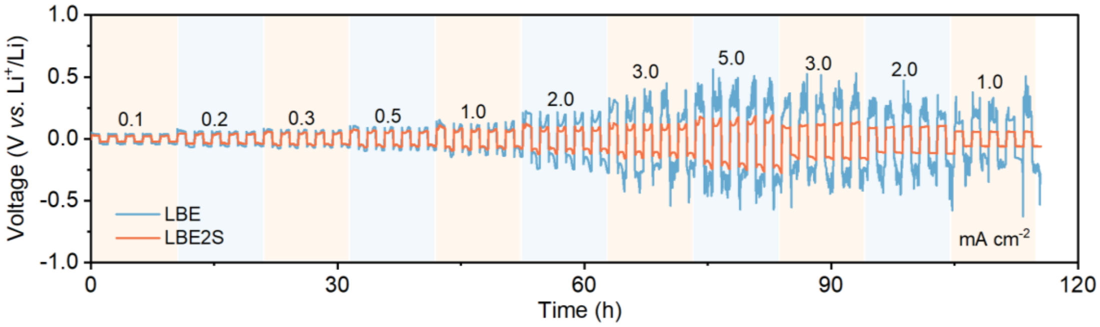

Figure S13. Rate performance of symmetrical cells with a fixed charge/discharge time of 1 h.

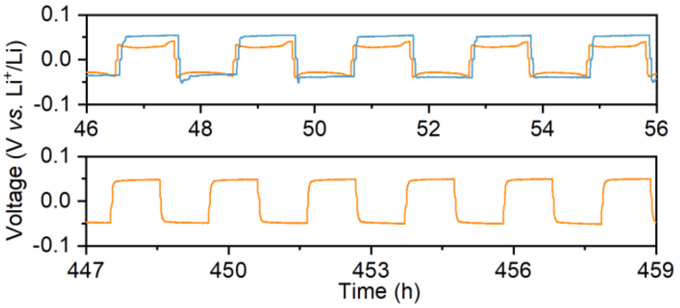

Figure S14. Enlarged figure of cycling performance of symmetrical cells at 1.0 mA cm^{-2} and 1.0 mAh cm^{-2}.

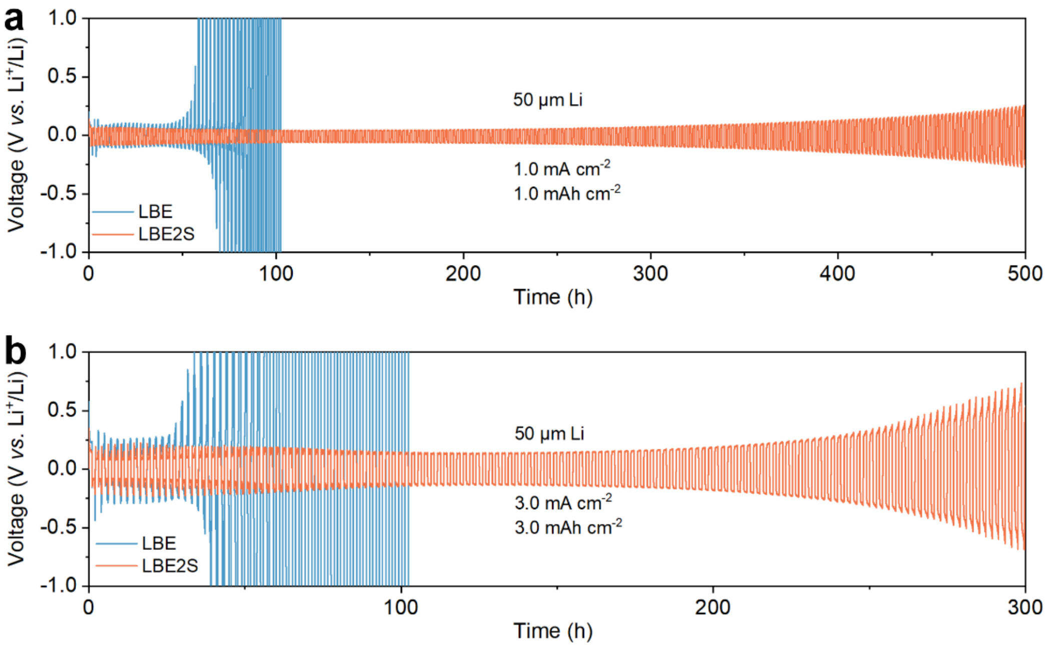

Figure S15. Electrochemical performance of Li/Li symmetric cells with 50 μm lithium metal foils cycled under (a) 1.0 mA cm^{-2} and (b) 3.0 mA cm^{-2} with a fixed cycling time of 1h.

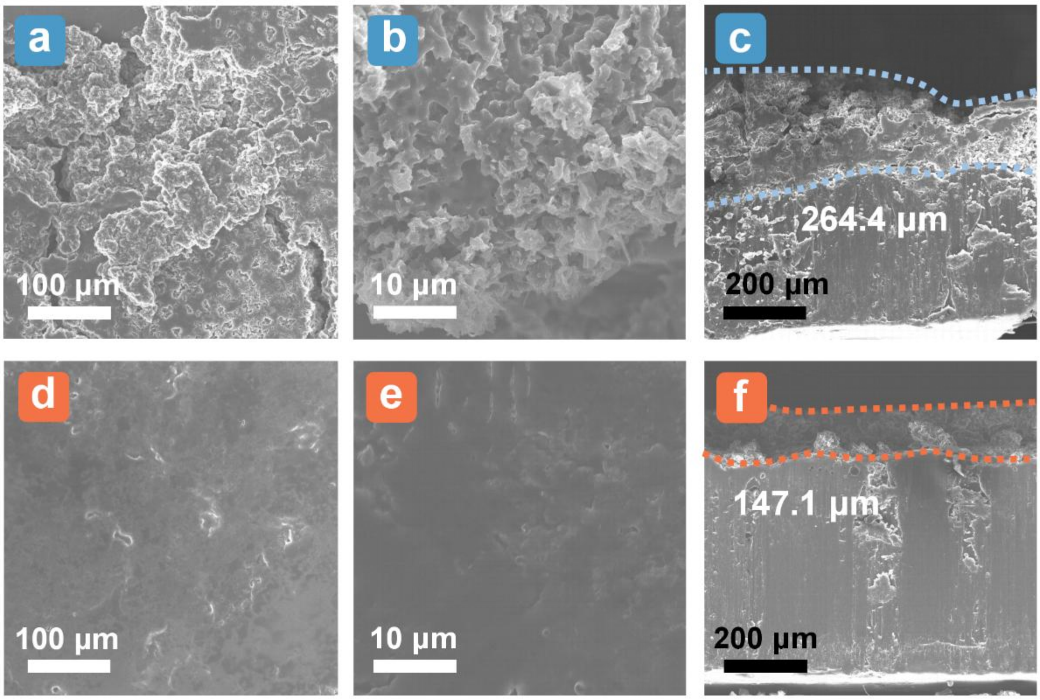

Figure S16. Lithium deposition morphology of lithium symmetrical cells cycled in (a-c) LBE and (d-f) LBE2S at 3.0 mA cm^{-2} and 3.0 mAh cm^{-2}.

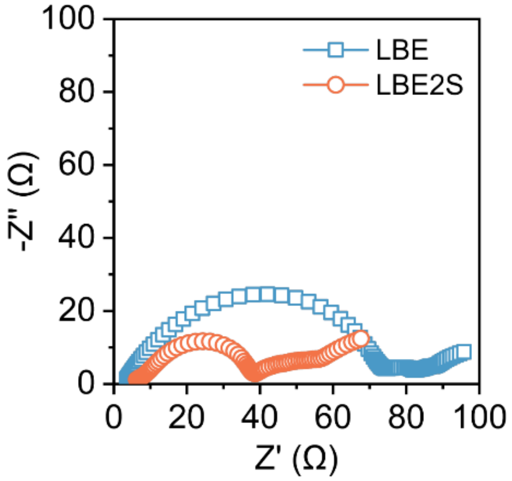

Figure S17. EIS of symmetrical cells after 20 cycles.

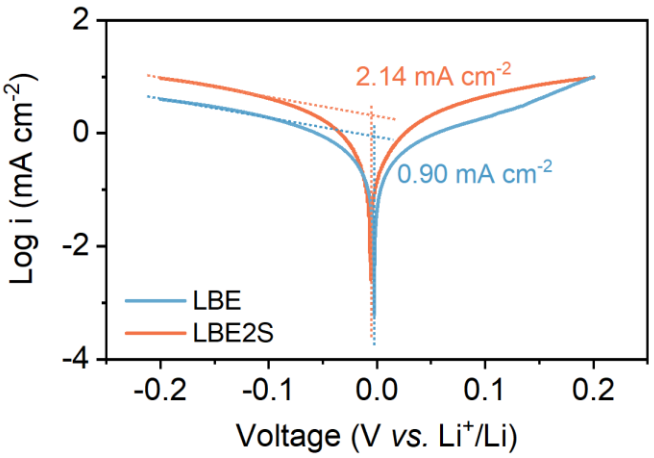

Figure S18. Tafel plot of symmetrical cells with LBE and LBE2S.

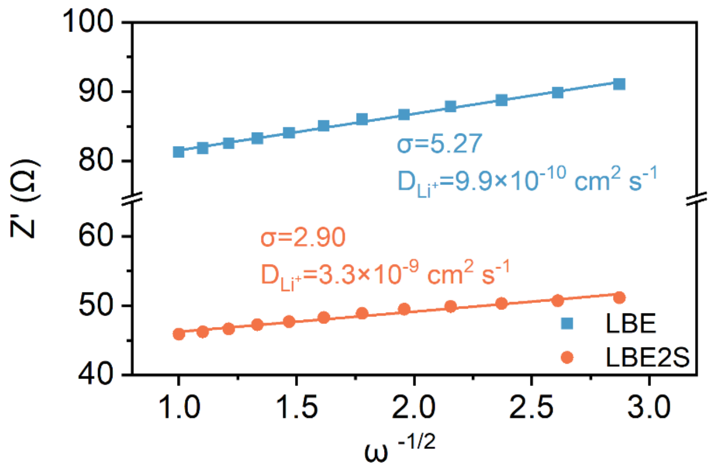

Figure S19. The calculated Li^+ diffusion coefficient from EIS spectra.

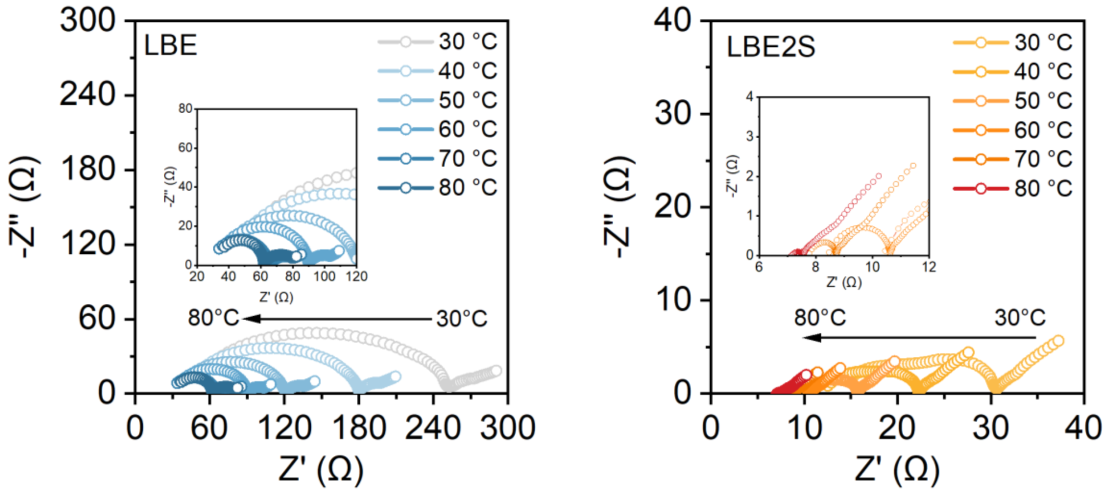

Figure S20. The calculated Li^+ diffusion coefficient from EIS spectra.

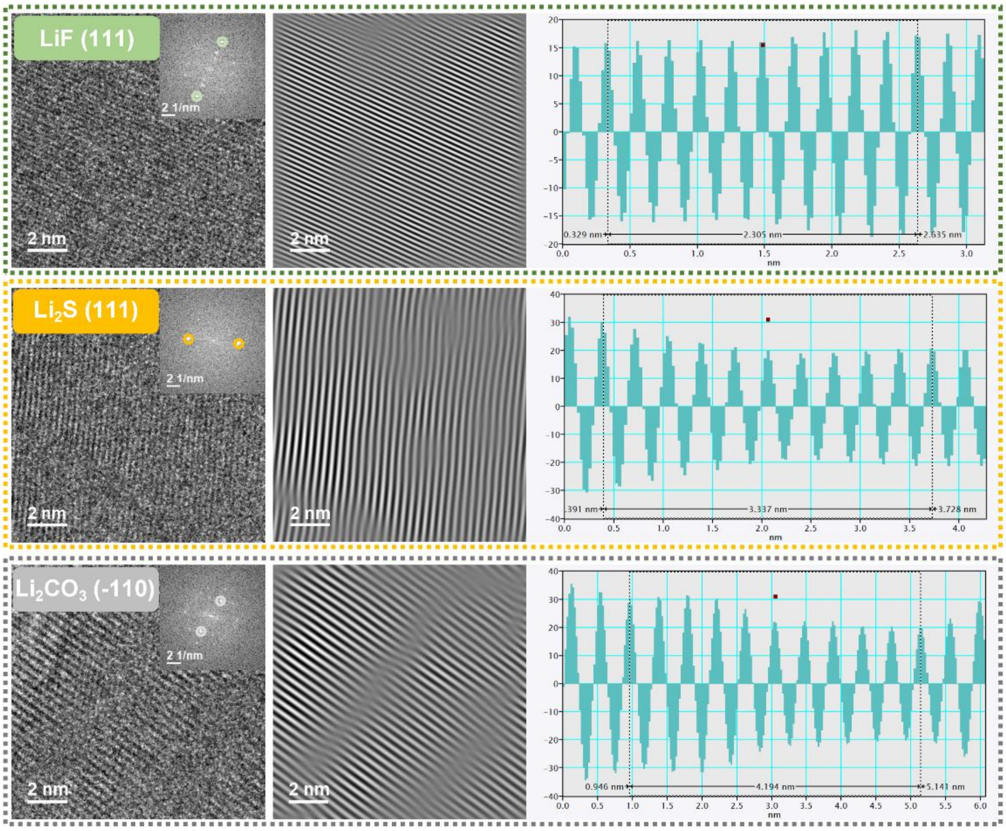

Figure S21. The IFFT images of cycled Li metal SEI in LBE2S electrolyte.

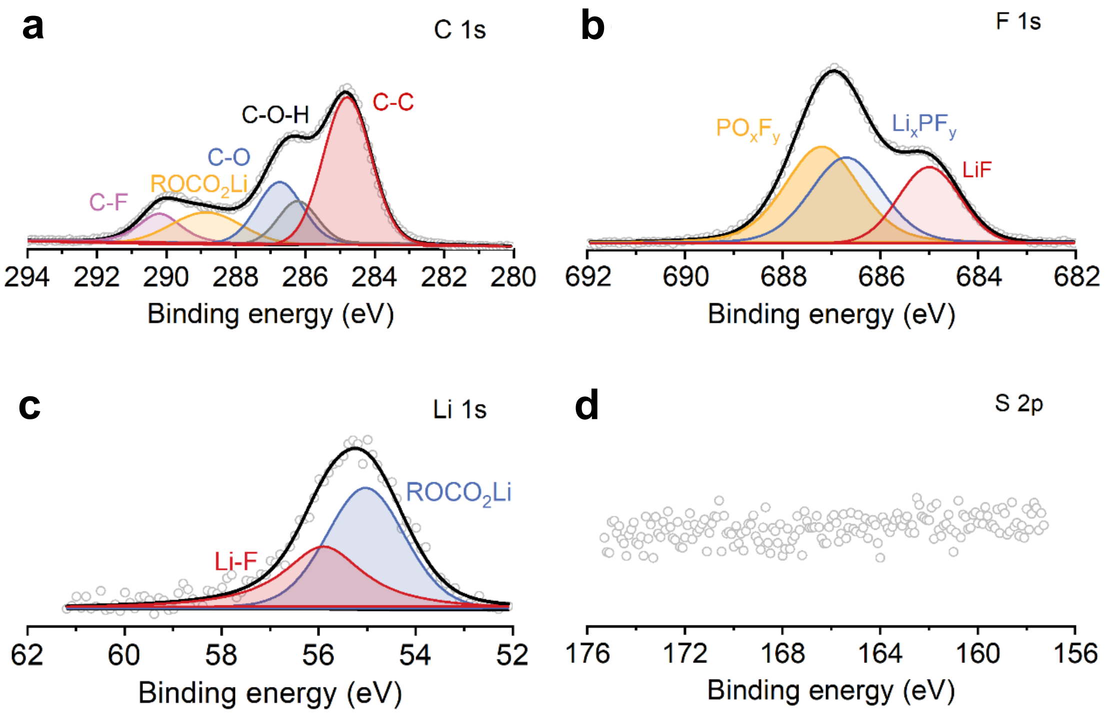

Figure S22. High-resolution XPS spectra of (a) C 1s, (b) F 1s, (c) Li 1s and (d) S 2p for the anode cycled in LBE.

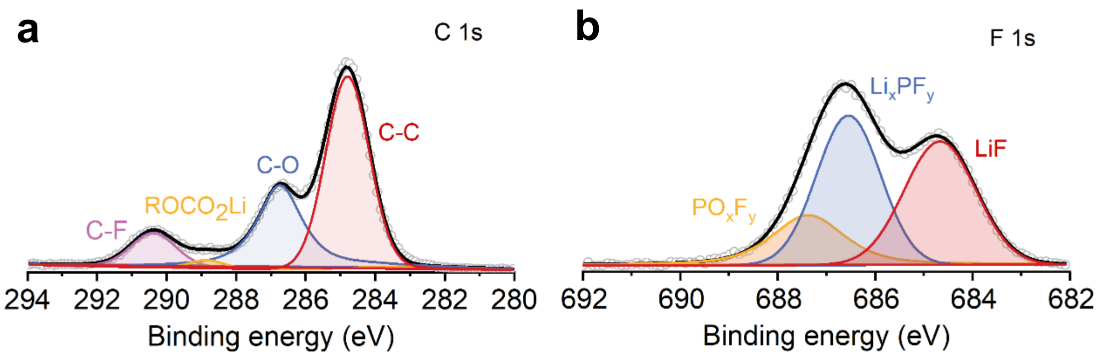

Figure S23. High-resolution XPS spectra of (a) C 1s and F (b) 1s for the anode cycled in LBE2S.

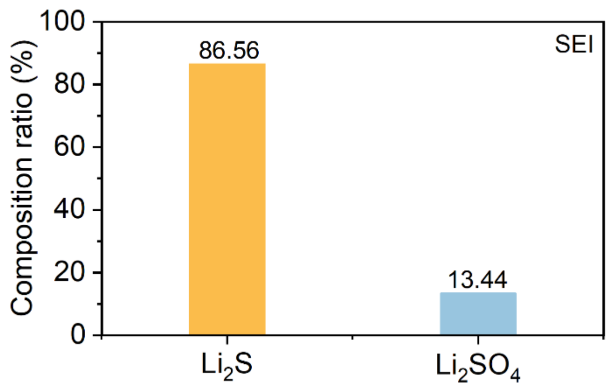

Figure S24. Corresponding composition ratio in XPS S 2p spectra for the anode cycled in LBE2S.

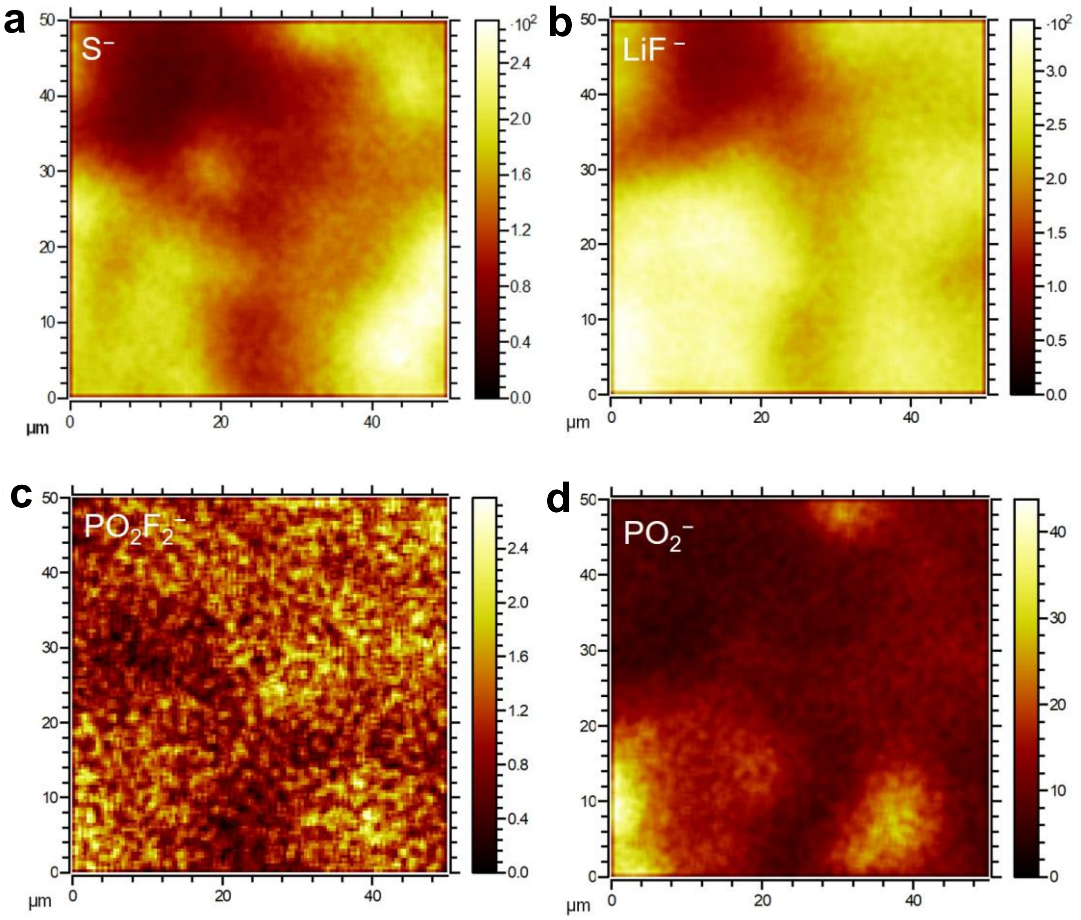

Figure S25. TOF-SIMS mappings of the S^-, LiF^-, PO_2^- and $PO_2F_2^-$ species on the surface of the cycled Li anode in LBE2S.

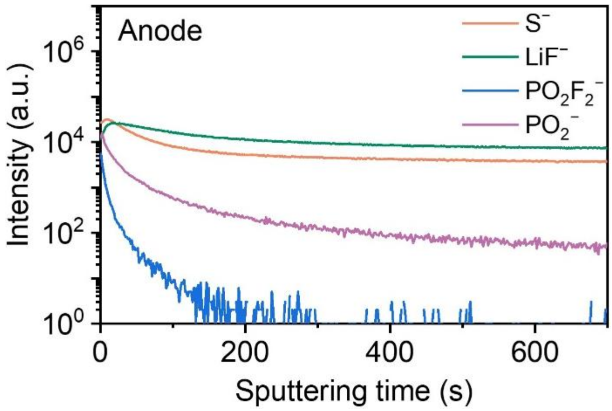

Figure S26. Depth profiling of several secondary ion fragments on Li anode in LBE2S.

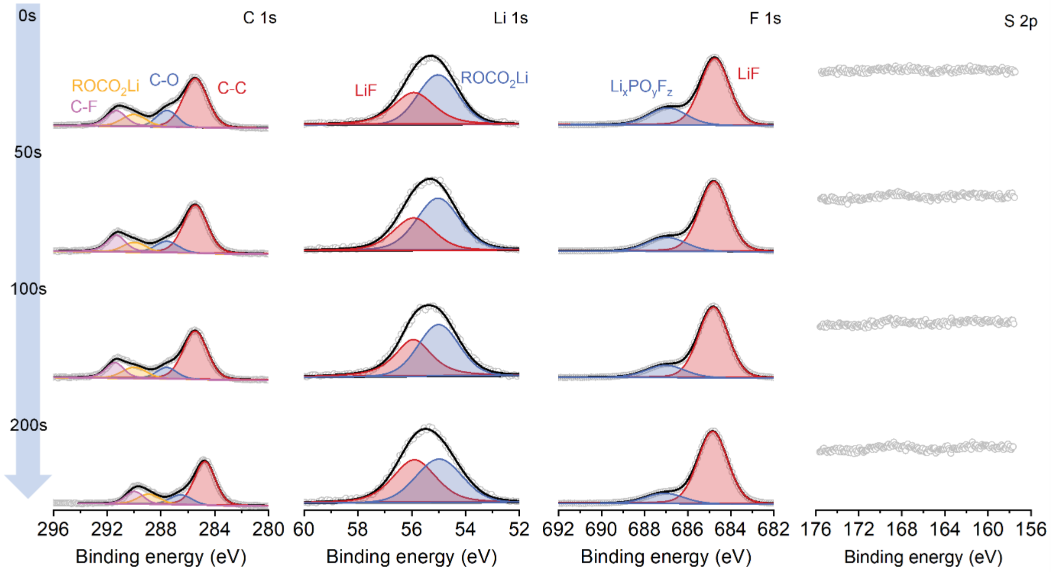

Figure S27. Etching XPS depth profiles of lithium metal anode after cycling for 100 cycles in LBE.

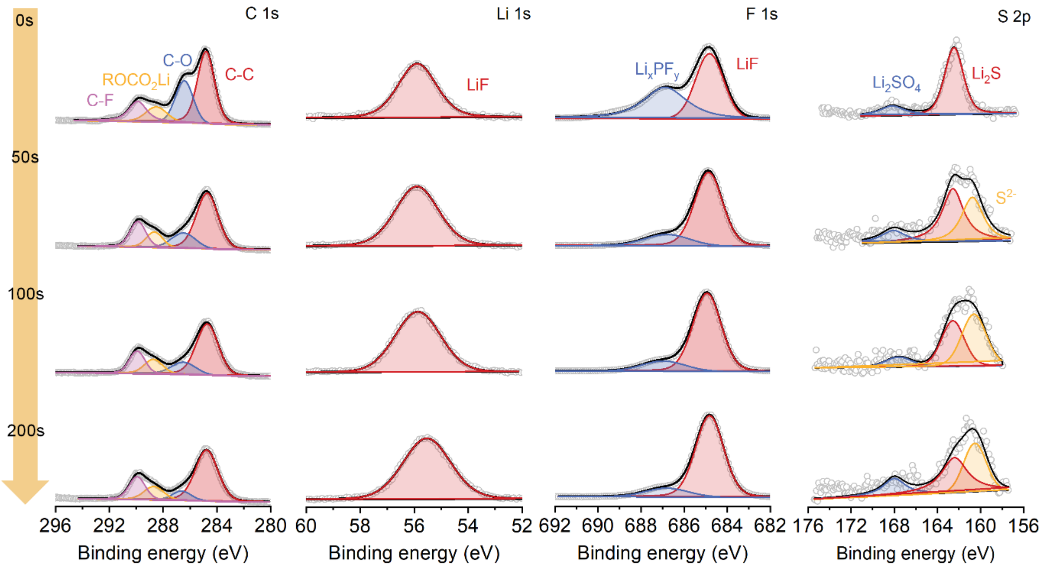

Figure S28. Etching XPS depth profiles of lithium metal anode after cycling for 100 cycles in LBE2S.

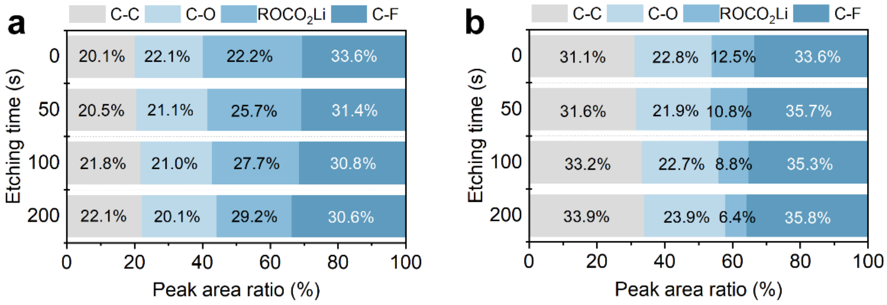

Figure S29. Comparison of the SEI composition ratio in (a) LBE and (b) LBE2S in C 1s XPS spectra.

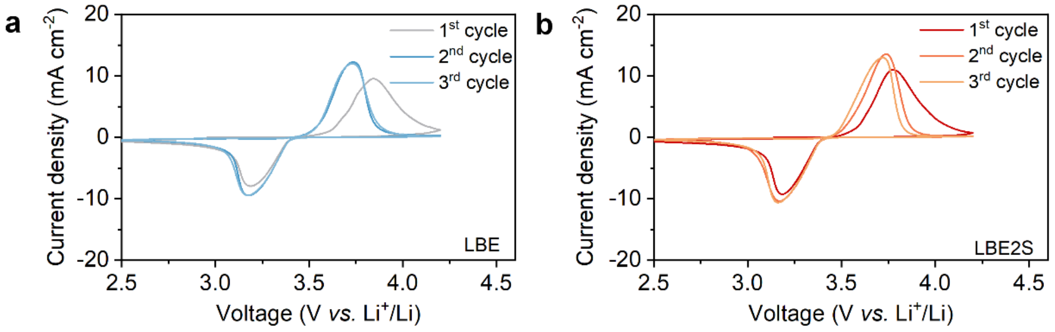

Figure S30. CV curves of LFP full cells for the first 3 cycles with (a) LBE and (b) LBE2S electrolyte, respectively.

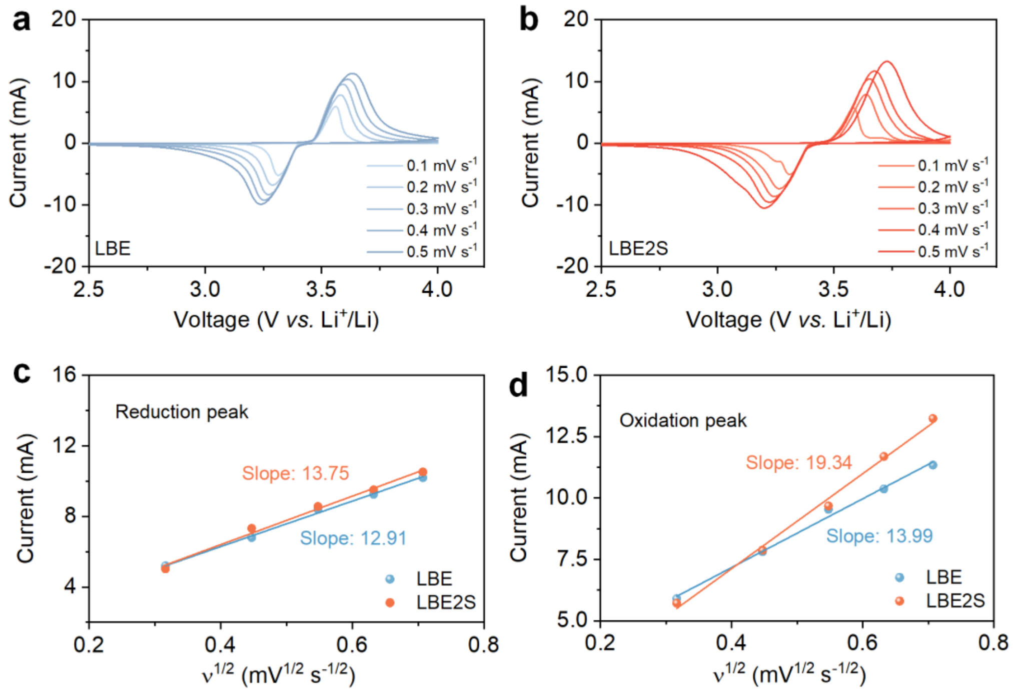

Figure S31. CV curves of the LFP full cells with LBE (a) and LBE2S (b) scanned at various scan rates. Linear relationship between the oxidation (c) and reduction peaks (d) and scan rates.

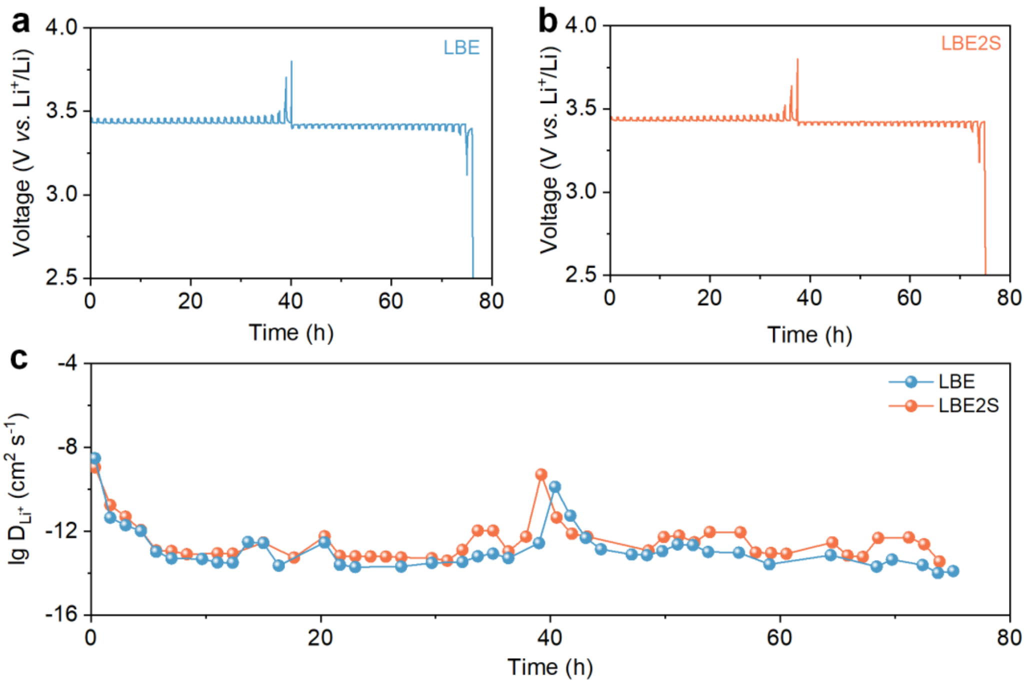

Figure S32. (a, b) The GITT curves and (c) the corresponding calculated Li^+ diffusion coefficient during delithiation and lithiation process.

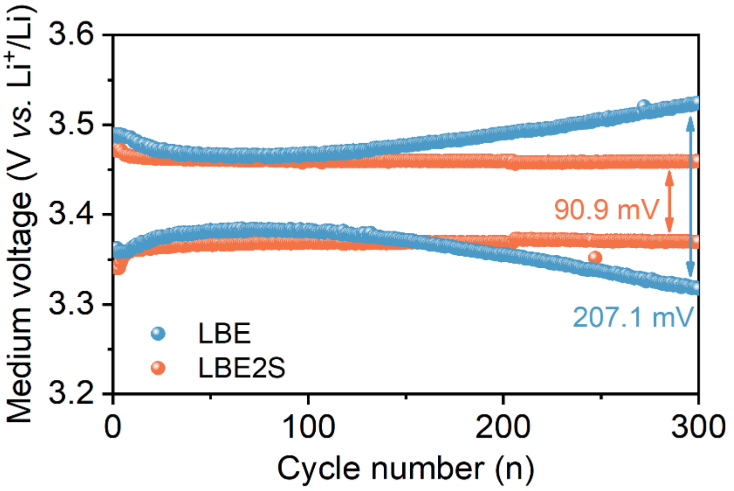

Figure S33. Charge/discharge overpotential evolution during 300 cycles.

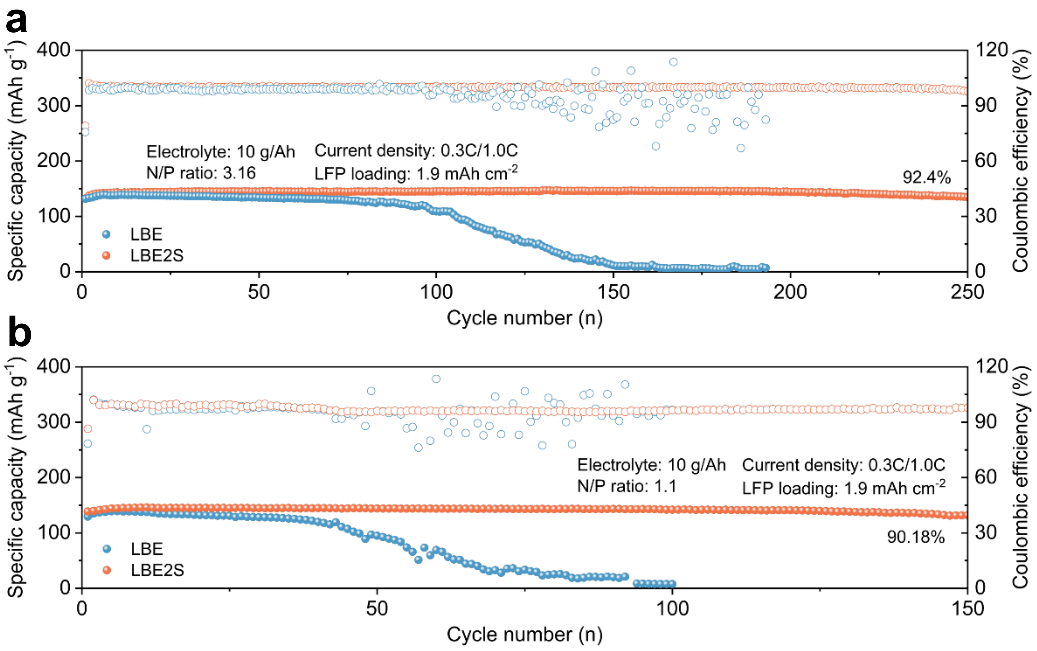

Figure S34. Cycling performance of Li||LFP full cells with a low N/P ratio (a) 3.16, (b) 1.05.

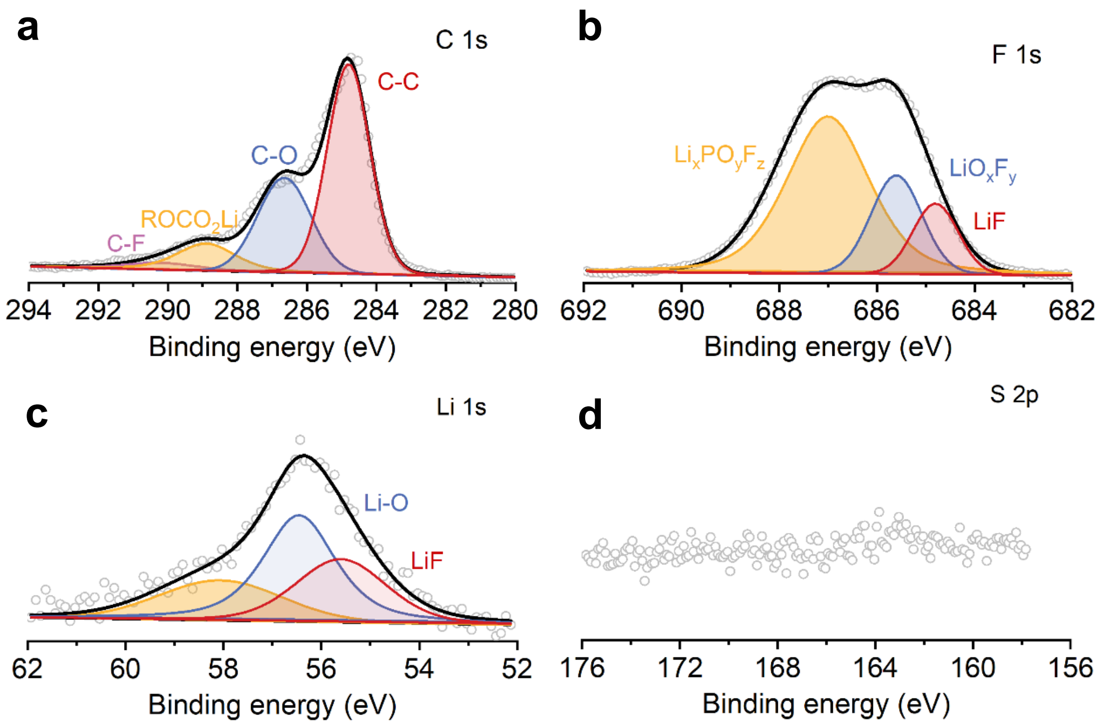

Figure S35. High-resolution XPS spectra of (a) C 1s, (b) F 1s, (c) Li 1s and (d) S 2p for the cathode cycled in LBE.

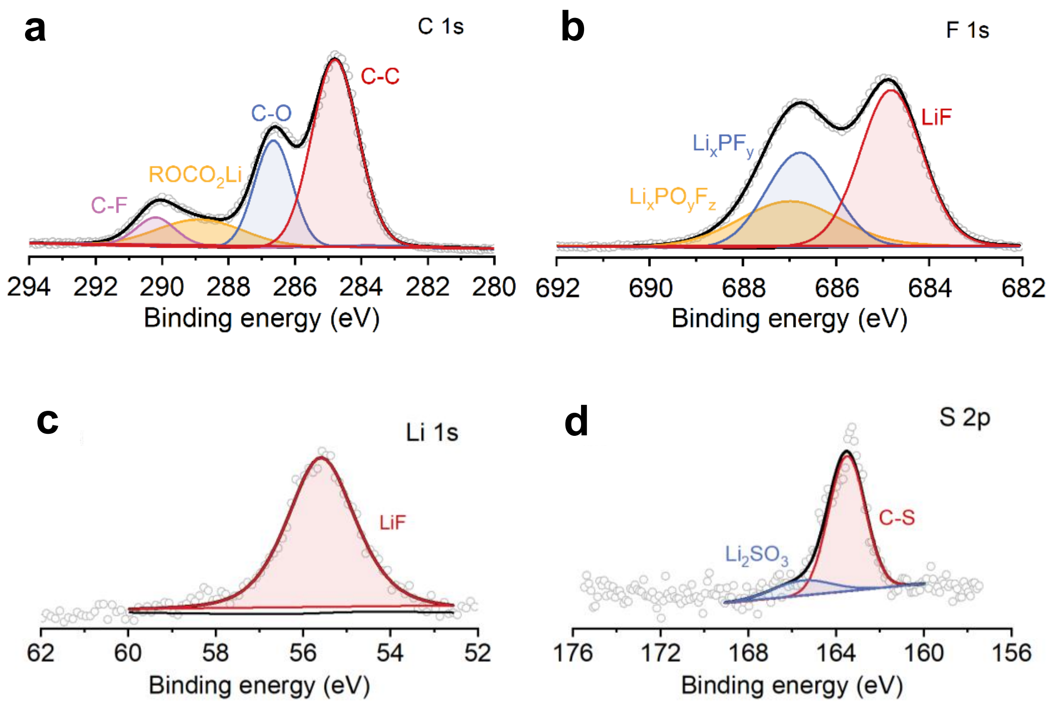

Figure S36. High-resolution XPS spectra of (a) C 1s, (b) F 1s, (c) Li 1s and (d) S 2p for the cathode cycled in LBE2S.

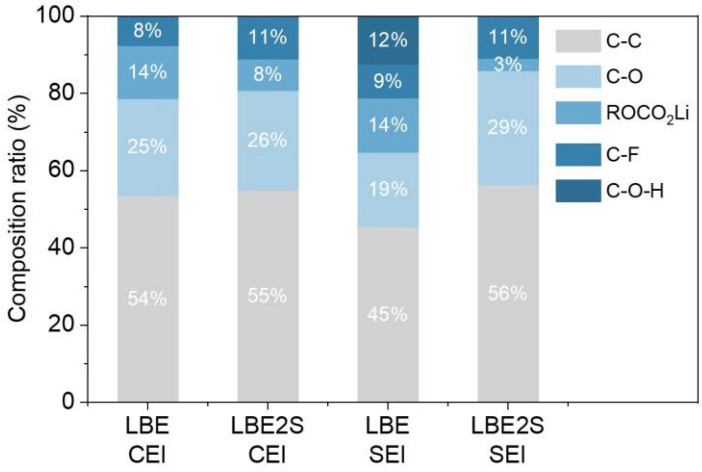

Figure S37. Comparison of the CEI and SEI composition ratio in LBE and LBE2S in C 1s XPS spectra.

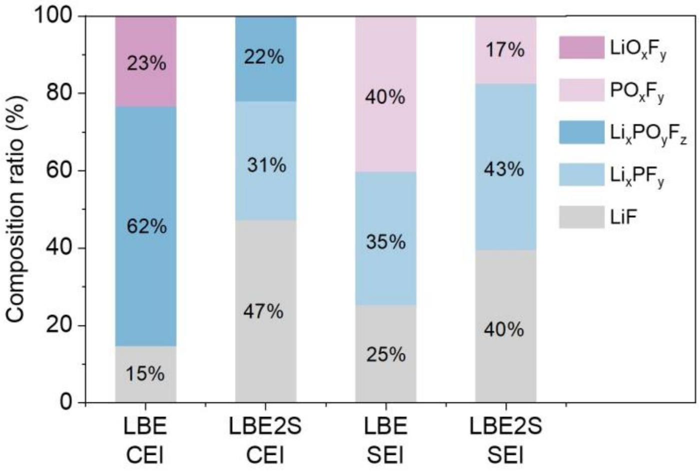

Figure S38. Comparison of the CEI and SEI composition ratio in LBE and LBE2S in F 1s XPS spectra.

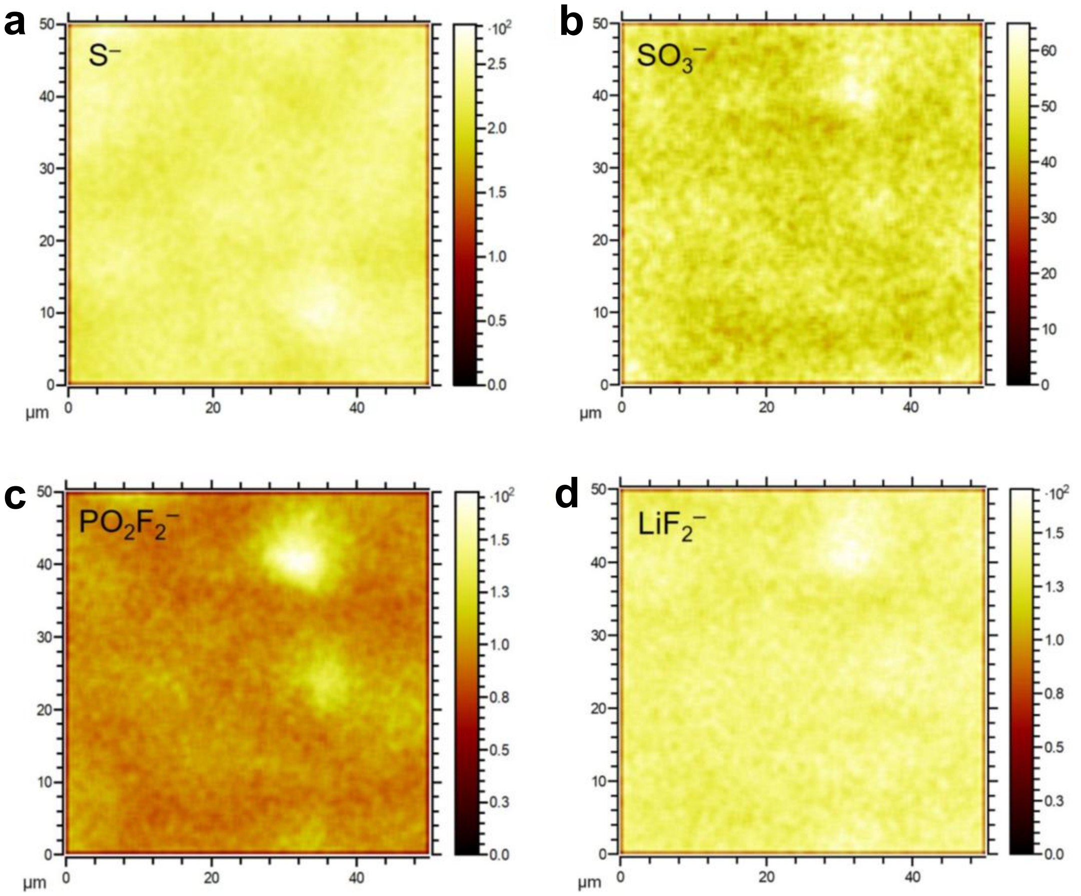

Figure S39. TOF-SIMS mappings of the S^-, SO_3^-, $PO_2F_2^-$ and LiF_2^- species on the surface of the cycled LFP cathode in LBE2S.

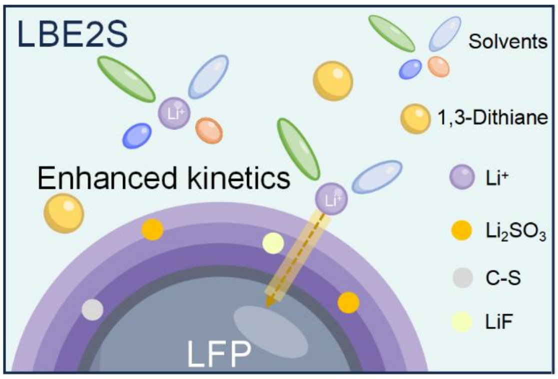

Figure S40. Schematic diagram of Li^+ migration on LFP in LBE2S.

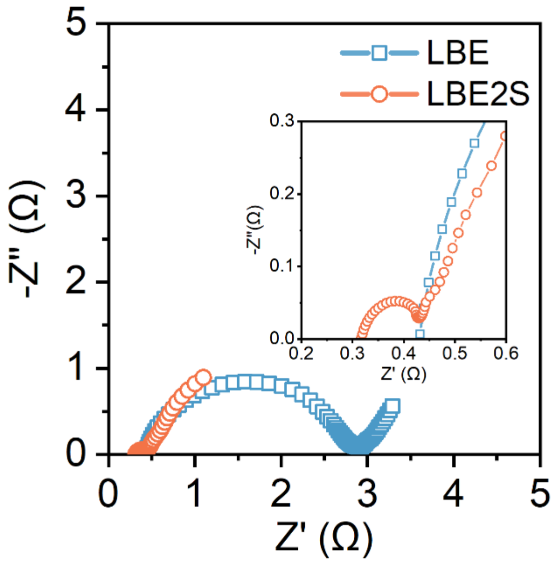

Figure S41. EIS spectra of pouch cell after 10 cycles.

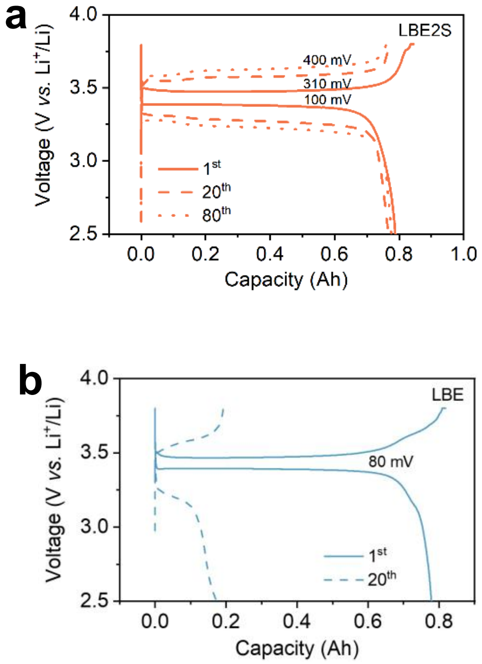

Figure S42. Charge/discharge voltage profiles of LFP pouch cell with (a) LBE2S and (b) LBE.

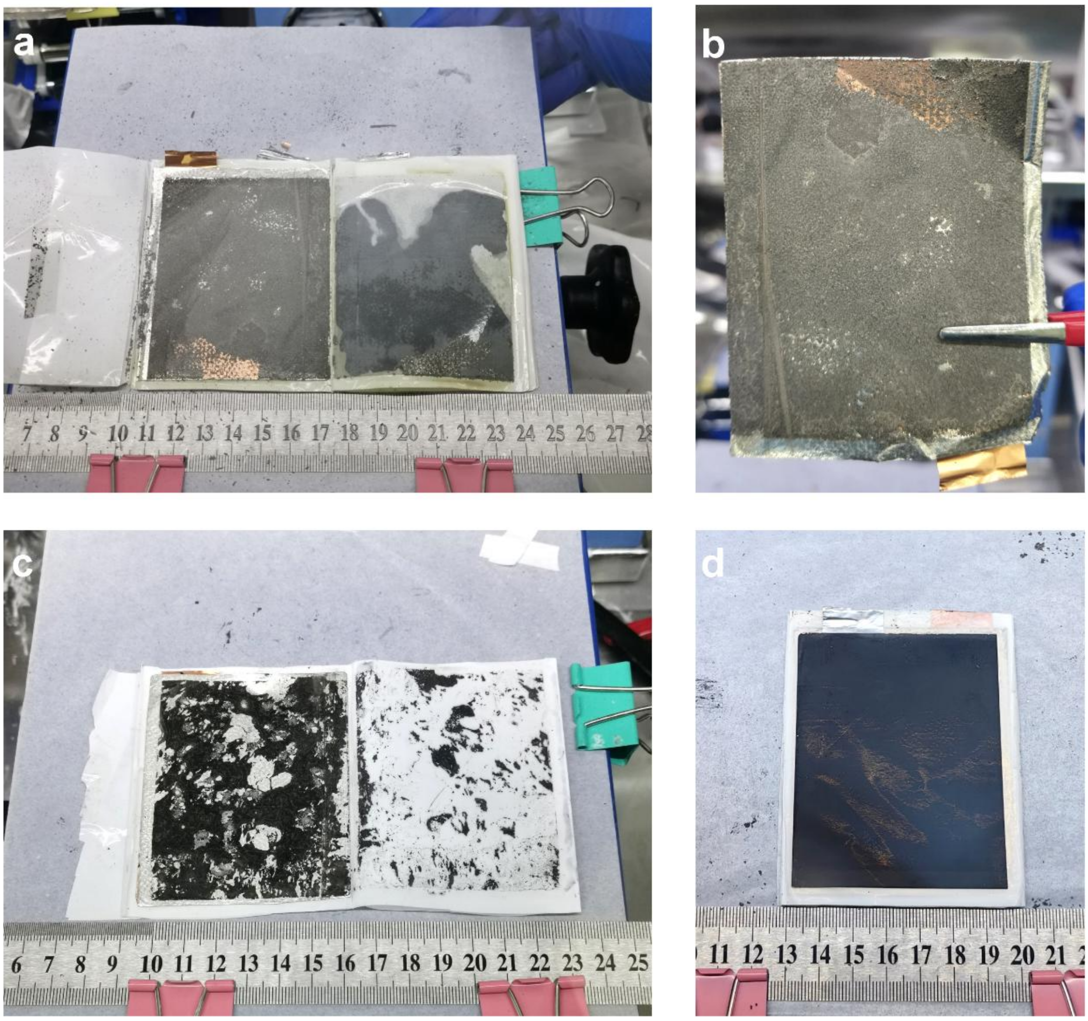

Figure S43. Photographs of electrodes disassembled from cycled pouch cell. (a) The anode in LBE after 10 cycles and the (b) corresponding enlarged image. (c) The anode and d cathode cycled in LBE2S after 10 cycles.

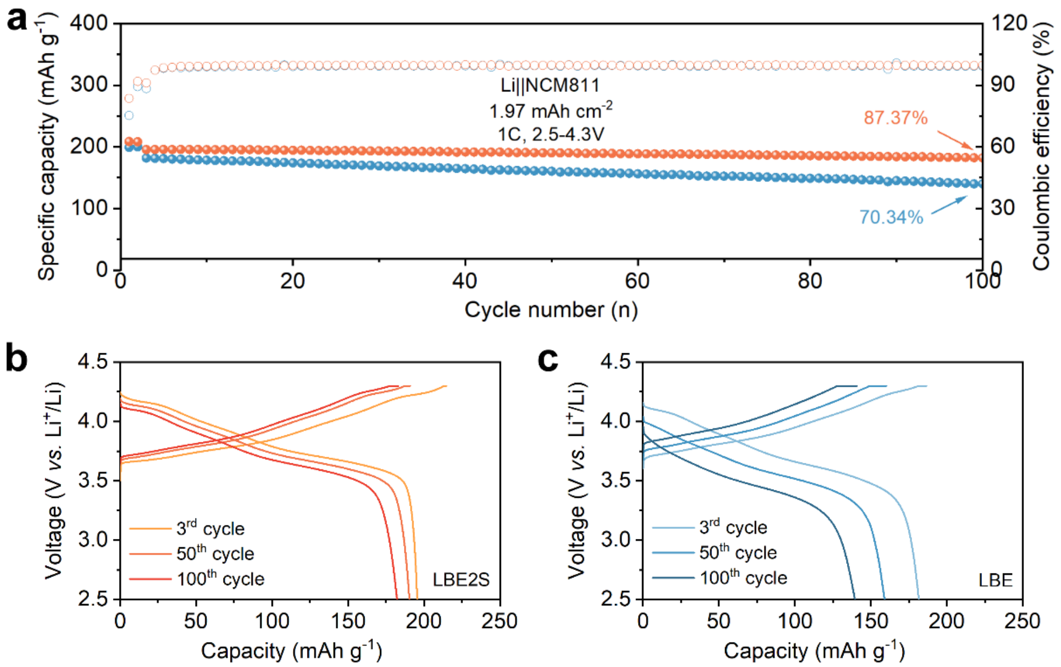

Figure S44. Electrochemical performance of Li//NCM811 full cells. (a) Cycling performance of Li//NCM811 full cells cycled at 1C. Corresponding charge/discharge voltage curves of Li//NCM811 full cells with (b) LBE2S and (c) LBE.

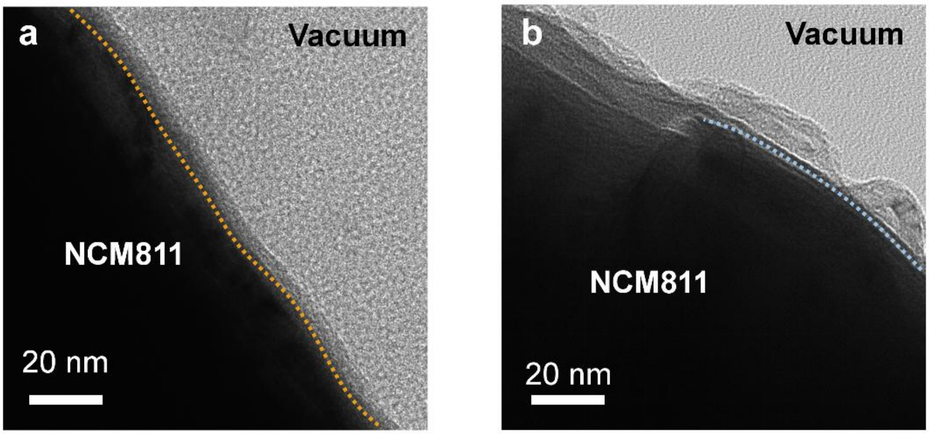

Figure S45. TEM images of NCM811 cathode cycled in (a) LBE2S and (b) LBE for 100 cycles.

Supplementary Tables

Table S1. S-content and molecular structure of representative sulfur-containing electrolyte additive.

**Category**	**Full name**	**Molecular structure**	**S-content ω (wt.%)**
**Thioether**	**1,3-Dithiane**	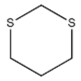	**53.50**
Sulfonate	Methylene methyl disulfonate	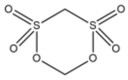	34.04
	1,3-Propane-sultone	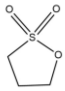	26.23
	Propargyl methane sulfonate	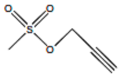	23.88
Sulfate	1,3,2-Dioxathiolane-2,2-dioxide	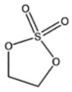	25.81
	1,3-Propanediolcyclic sulfate	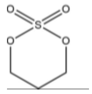	23.19
	1,3,2-Benzodioxathiole 2,2-dioxide	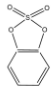	18.60
Sulfite	Ethylene sulfite	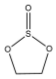	29.63
	Propylene sulfite	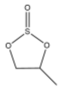	26.23
	Dimethyl sulfite	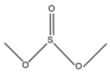	29.09
Sulfone	Sulfolane	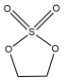	26.67
	Methyl 2,2-difluoro-2-(fluorosulfonyl) acetate	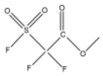	16.67
	p-Toluenesulfonyl isocyanate	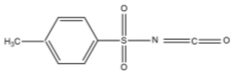	16.24

The S-content ω_S was calculated according to Equation 2:

$$\omega_S = \frac{32.07 \times n}{\mathrm{M}} \tag{2}$$

Here, n represents the number of sulfur atoms and M represents the molecular mass in the corresponding molecular.

Table S2. The calculated electrophilicity and nucleophilicity of 1,3-dithiane element.

Atom	Electrophilic(f(-))	Nucleophilic(f(+))
C1	−0.037	−0.048
C2	−0.027	−0.031
C3	−0.03	−0.029
C4	−0.036	−0.027
S1	0.231	0.280
S2	0.402	0.303
H1	0.071	0.087
H2	0.061	0.100
H3	0.063	0.063
H4	0.056	0.058
H5	0.033	0.037
H6	0.076	0.082
H7	0.065	0.054
H8	0.072	0.070

Table S3. Calculated charge on the carbon atoms of EC, DMC and EMC.

Carbon in EC [a]	Charge -1	Charge -2	Carbon in DMC [b]	Charge -1	Charge -2	Carbon in EMC [c]	Charge -1	Charge -2
C_1	0.722	0.259	C_2	0.723	0.255	C_3	0.717	0.260
C_2 and C_3	-0.260	-0.036	C_1 and C_3	-0.109	0.016	C_1	-0.119	-0.503
						C_2	0.005	-0.091
						C_4	-0.034	-0.260

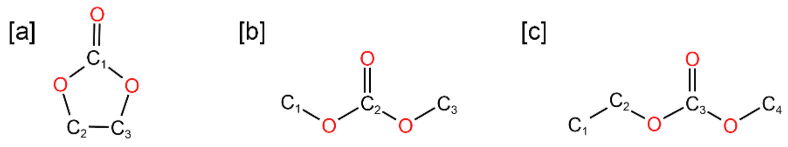

Table S4. Fitting equivalent circuit model and impedance parameters of Li//Li symmetrical cells.

Li//Li symmetrical cells	R_{ct} (Ω)	R_{SEI} (Ω)
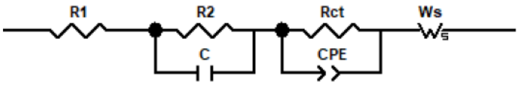	20th	
LBE	17.16	73.75
LBE2S	11.42	32.11

Table S5. Comparison of this work with reported electrolytes for LFP full cells.

Strategy	Electrolyte	Testing step	Cycling performance	References
1,3-Dithiane additive	1M $LiPF_6$ in EC: DMC: EMC (1:1:1, w: w: w) with 2 wt. % of 1,3-dithiane	2.5-3.8V,1C	83.6%, 3000 cycles	This work
Synergistic additives of $LiNO_3$ and ethylene sulfite (ES)	1 M LiTFSI in DOL: DME (v: v = 1: 1) with 2 wt.% $LiNO_3$ and 1 wt.% ES	2.4-3.8V, 0.2C C-0.5C D	89.7%, 150 cycles	[1]
NO_3^- intercalated LiAl layered double hydroxides (LiAl-LDHs) additive	1 M $LiPF_6$ in EC: DEC (1: 1, v: v) with 10 mg mL^{-1} LiAl-LDHs	2.5-4.2 V, 1C	90%, 800 cycles	[2]
Liquid alloy GaSnIn additive	1 M $LiPF_6$ in EC: DEC (1: 1, v: v) with 1 mg mL^{-1} GaSnIn	2.5-4.0 V, 1C	72.5%, 2500 cycles	[3]
Lithium bis(fluorosulfonyl)imide additive (LiFSI)	0.45 M $LiPF_6$ +0.05 M LiFSI-DEC/EC	2.7-4.2 V, 0.5C	86.6%, 100 cycles	[4]
2,2,2-Trifluoroethyl trifluoroacetate (2,2,2-TFTF) additive	1 M LiTFSI in DOL: DME (1: 1, v: v) with 1 wt.% $LiNO_3$ and 4% 2,2,2-TFTF	2.4-4.2 V, 1C	86%, 500 cycles	[5]
$LiNO_3$ single lithium salt	1.5 M $LiNO_3$ in TEP: FEC (3:1)	2.5-4.2V, 1C	96.39%, 1000 cycles	[6]
Dual-salt (LiFSI, $LiNO_3$)	2 mmol LiFSI and 0.25 mmol $LiNO_3$ in DOL/DME	3-3.8V, 1C	90%, 300cycles	[7]
1,3-propanesultone additive with FEC solvent	1M $LiPF_6$ in EA: FEC (10: 1, v: v) with 2 vt. % of 1,3-propanesultone (PS)	2.5-4.0V, 0.5C	88%, 300cycles	[8]
1,2-difluorobenzene (1,2-dfBen) dilutant	2 M LiFSI in Pyr13FSI: 1,2-dfBen (1: 1, v: v)	2.4-4.0V, 2C	96%, 300 cycles	[9]
(3,3,3-trifluoropropyl) trimethoxysilane (TFTMS) fluorinated siloxane solvent	1.5 M LiFSI in DME: FEC: TFTMS (1: 2: 2, v: v: v)	2.5-4.0V, 2C	76.5%, 600 cycles	[10]
High-concentration electrolyte	3.5 M LiTFSI +0.05 M $LiPF_6$ in DMC	2.0-4.0V, 1C	107.8%, 100 cycles	[11]
Electrolyte components proportion optimization	1.16M $LiPF_6$ in TEP: EMC: EC (2: 4: 1.5, n: n: n)	2.8-3.8V, 1C	92.6%, 100cycles	[12]

Table S6. Fitting equivalent circuit model and impedance parameters of Li//LFP pouch full cells.

**Li//LFP pouch cells**	**R_{ct} (Ω)**
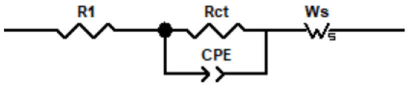	10th
LBE	2.39
LBE2S	0.12

REFERENCES

[1] Lin Y, Chen J, Zhang H et al. *In-situ* construction of high-mechanical-strength and fast-ion-conductivity interphase for anode-free Li battery. *J. Energy Chem.* 2023; **80**: 207-214.

[2] Li B, Cai Q, Zhang J et al. LiAl-LDHs additives in carbonate electrolytes for realizing dendrite-free lithium deposition in Li metal batteries. *Chem. Eng. J.* 2025; **509**: 161322.

[3] Xu W, Liao X, Xu W et al. Gradient SEI layer induced by liquid alloy electrolyte additive for high rate lithium metal battery. *Nano Energy* 2021; **88**: 106237.

[4] Quan Y, Li S, Zhang N et al. Improving performances of cathode-electrolyte interphase via the potentiostatic reduction of lithium bis(fluorosulfonyl)imide additive. *Electrochim. Acta* 2023; **460**: 142617.

[5] Yang S, Hao M, Wang Z et al. 2,2,2-Trifluoroethyl trifluoroacetate as effective electrolyte additive for uniform Li deposition in lithium metal batteries. *Chem. Eng. J.* 2022; **435**: 134897.

[6] Liao C, Han L, Wang W et al. Non-Flammable Electrolyte with Lithium Nitrate as the Only Lithium Salt for Boosting Ultra-Stable Cycling and Fire-Safety Lithium Metal Batteries. *Adv. Funct. Mater.* 2023; **33(17)**: 2212605.

[7] Zheng L, Hou R, Shi T et al. Reversing nitride/fluoride distribution in the solid electrolyte interphase enables a highly reversible lithium metal anode. *Nano Energy* 2025; **138**: 110849.

[8] Li Y, Wen B, Li N et al. Electrolyte Engineering to Construct Robust Interphase with High Ionic Conductivity for Wide Temperature Range Lithium Metal Batteries. *Angew. Chem. Int. Ed.* 2025; **64(2)**: e202414636.

[9] Tu H, Li L, Wang Z et al. Tailoring Electrolyte Solvation for LiF-Rich Solid Electrolyte Interphase toward a Stable Li Anode. *ACS Nano* 2022; **16(10)**: 16898-16908.

[10] Huang G, Liao Y, Liu H et al. Electrolyte Engineering via Fluorinated Siloxane Solvent for Achieving High-Performance Lithium-Metal Batteries. *ACS Nano* 2024; **18(24)**: 15802-15814.

[11] Li Y, Wang Z, Lin W et al. A Concentrated Electrolyte of LiTFSI and Dimethyl Carbonate for High-Voltage Li Batteries. *ACS Appl. Energy Mater.* 2023; **6(18)**: 9337-9346.

[12] Liu M, Liu W, Zeng Z et al. Chelating Solvent Mediated Solvation Structure Enables High-Rate Operation of Ah-Level Li-Ion Batteries in Nonflammable Phosphate Electrolyte. *Adv. Energy Mater.* 2025; **n/a(n/a)**: 2500864.
